# SARS-CoV-2 3CL^pro^ mutations selected in a VSV-based system confer resistance to nirmatrelvir, ensitrelvir, and GC376

**DOI:** 10.1126/scitranslmed.abq7360

**Published:** 2022-10-04

**Authors:** Emmanuel Heilmann, Francesco Costacurta, Seyed Arad Moghadasi, Chengjin Ye, Matteo Pavan, Davide Bassani, Andre Volland, Claudia Ascher, Alexander Kurt Hermann Weiss, David Bante, Reuben S. Harris, Stefano Moro, Bernhard Rupp, Luis Martinez-Sobrido, Dorothee von Laer

**Affiliations:** ^1^ Institute of Virology, Medical University of Innsbruck, Innsbruck, 6020, Austria.; ^2^ Department of Biochemistry, Molecular Biology and Biophysics, Institute for Molecular Virology, University of Minnesota, Minneapolis, MN 55455, United States.; ^3^ Texas Biomedical Research Institute, San Antonio, TX 78229, USA.; ^4^ Molecular Modeling Section (MMS), Department of Pharmaceutical and Pharmacological Sciences, University of Padua, Via F. Marzolo 5, 35131, Padova, Italy.; ^5^ Institute for Biomedical Aging Research, University of Innsbruck, Innsbruck, 6020, Austria.; ^6^ Department of Biochemistry and Structural Biology, University of Texas Health San Antonio, San Antonio, TX 78229, United States.; ^7^ Howard Hughes Medical Institute, University of Texas Health San Antonio, San Antonio, TX 78229, United States.; ^8^ Division of Genetic Epidemiology, Medical University of Innsbruck, Innsbruck, 6020, Austria.; ^9^ k.-k. Hofkristallamt, San Diego, CA 92084, United States.

## Abstract

Protease inhibitors are among the most powerful antiviral drugs. Nirmatrelvir is the first protease inhibitor against the SARS-CoV-2 protease 3CL^pro^ that has been licensed for clinical use. To identify mutations that confer resistance to this protease inhibitor, we engineered a chimeric vesicular stomatitis virus (VSV) that expressed a polyprotein composed of the VSV glycoprotein G, the SARS-CoV-2 3CL^pro^, and the VSV polymerase L. Viral replication was thus dependent on the autocatalytic processing of this precursor protein by 3CL^pro^ and release of the functional viral polymerase L, and replication of this chimeric VSV was effectively inhibited by nirmatrelvir. Using this system, we applied nirmatrelvir to select for resistance mutations. Resistance was confirmed by retesting nirmatrelvir against the selected mutations in an additional VSV-based systems, in an independently developed cellular system, in a biochemical assay, and in a recombinant SARS-CoV-2 system. We demonstrate that some mutants are cross-resistant to ensitrelvir and GC376, whereas others are less resistant to these compounds. Furthermore, we found that most of these resistance mutations already existed in SARS-CoV-2 sequences that have been deposited in the NCBI and GISAID databases, indicating that these mutations were present in circulating SARS-CoV-2 strains.

## Teaser

Mutations in the SARS-CoV-2 main protease that conferred resistance to the protease inhibitor nirmatrelvir were identified using a VSV-based system.

## INTRODUCTION

In late 2019, the zoonotic transmission of a new coronavirus, severe acute respiratory syndrome coronavirus 2 (SARS-CoV-2), into the human population (*1*), has led to worldwide efforts to find effective treatments against the various pathologies caused by the virus. Inhibitors of viral enzymes, such as proteases, have proven to be highly potent drugs in the treatment of HIV and Hepatitis C virus infections. However, resistant viruses rapidly emerge unless the protease inhibitors are given in combination with other directly acting antivirals (*2*, *3*). SARS-CoV-2 encodes two proteases. The 3-Chymotrypsin-like protease (3CL^pro^) cleaves 11 sites in the viral polyproteins pp1a and pp1ab and is also referred to as the main protease (M^pro^) or non-structural protein 5 (nsp5), indicating that it cleaves more sites than the second protease and its location within the polyproteins, respectively (*4*). The second viral protease, Papain-like protease (PL^pro^) cleaves three additional sites in pp1a and pp1ab (*5*). Thus, both proteases are essential for viral replication and therefore interesting drug targets.

Recently, the 3CL^pro^ inhibitor nirmatrelvir was approved for clinical use. Nirmatrelvir acts as a peptidomimetic, covalent inhibitor binding to the catalytic site cysteine (C145), thereby blocking its function (*6*–*8*). Nirmatrelvir has been authorized in combination with ritonavir by the U.S. food and drug administration (FDA) for emergency use in high-risk SARS-CoV-2-infected individuals under the trade name Paxlovid (EUA 105 Pfizer Paxlovid, 22.12.2021). In the studies leading to the Paxlovid (nirmatrelvir/ritonavir) emergency use authorization (EUA), mouse hepatitis virus (MHV) 3CL^pro^ was used as a surrogate for SARS-CoV-2 3CL^pro^ to generate resistance data, which may not be the ideal system. In addition, very recently several preprints have described nirmatrelvir resistance mutations in authentic SARS-CoV-2, either generated de novo (*9*, *10*), found in isolates (*11*, *12*), or modelled in silico (*13*). Working with SARS-CoV-2 requires biosafety level 3 (BSL-3) installations due to its virulence (*14*). Even more so, performing SARS-CoV-2 antibody or antiviral resistance studies demand utmost caution to avoid biosafety breaches and subsequent spread of mutant variants.

To address these caveats, we describe in this study a BSL-2 system based on VSV that allows the selection of resistance mutations in the SARS-CoV-2 3CL^pro^. Several mutations identified were validated in cell-based, biochemical, and recombinant SARS-CoV-2 assays and two mutations were found to be identical to (L167F) or at the same residue (Q192) as those described in the other manuscripts characterizing resistance in authentic SARS-CoV-2. We furthermore showed that some mutations selected by one 3CL^pro^ inhibitor can confer cross-resistance to other inhibitors. In contrast, other mutations appeared more inhibitor-specific. We attributed these effects to the distinct chemical structures of the inhibitors and occupation within the active site of the 3CL^pro^. Lastly, we modelled catalytic site mutations with Robetta (*15*) and Molecular Operating Environment (*16*) to elucidate their mechanism of resistance.

## RESULTS

### MHV 3CL^pro^ is less sensitive to nirmatrelvir than SARS-CoV-2 3CL^pro^

**Fig. 1.**
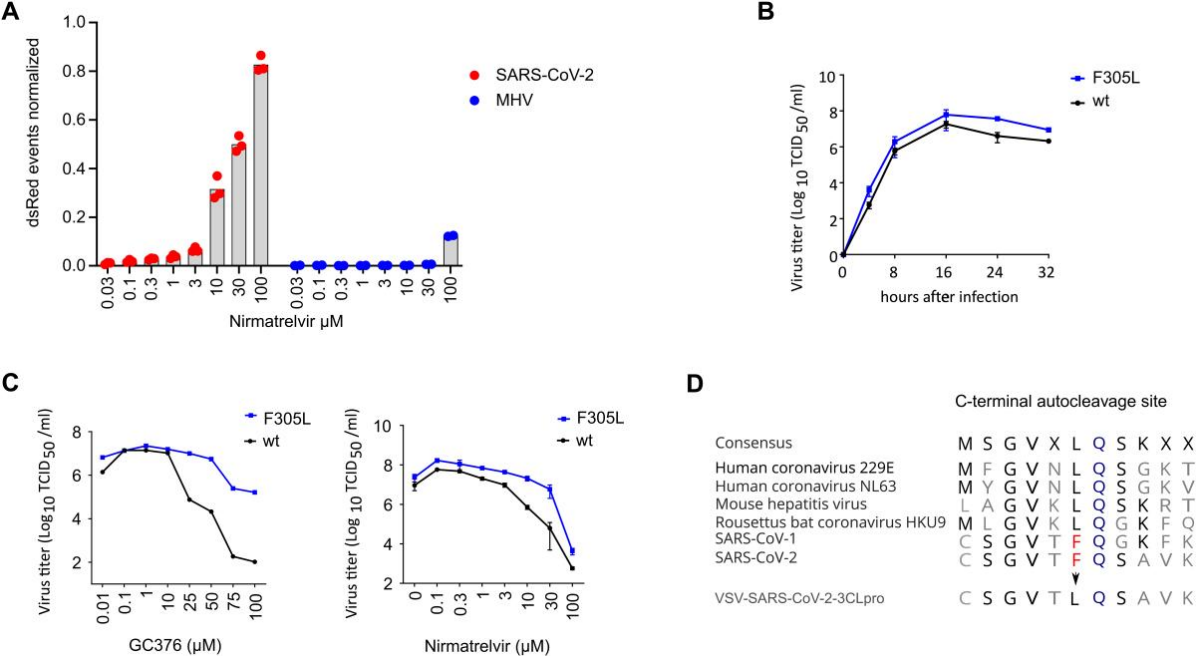
A VSV-based non-gain-of-function system was developed to predict SARS-CoV-2 3CL^pro^ mutations. **(A)** 3CL^pro^ from SARS-CoV-2 and mouse hepatitis virus (MHV) were tested in a gain-of-signal assay. Data are presented as individual points of n = 3 biologically independent replicates per condition for SARS-CoV-2 3CL^pro^ and n = 2 for MHV 3CL^pro^, average values are represented by histogram bars. **(B)** Replication kinetics are shown for wild-type (wt) VSV-G-3CL^pro^-L and GC376-selected F305L variant. Data are presented as SD of n = 2 biologically independent replicates per condition. **(C)** GC376 and nirmatrelvir dose responses are shown for wild-type (wt) VSV-G-3CL^pro^-L and GC376-selected F305L variant. Data are presented as means of n = 2 (GC376) and n = 3 (Nirmatrelvir) biologically independent replicates per condition. **(D)** Sequence alignment of C-terminal autocleavage sites is shown for SARS-CoV-2 3CL^pro^ and related coronaviruses.

We compared the sensitivity of SARS-CoV-2 and MHV 3CL^pro^ to the active component of Paxlovid, nirmatrelvir, using the gain-of-signal variant of a VSV-based 3CL^pro^ measurement assay shown in **fig. S1A and B** and described in detail recently (*17*). In brief, the coronavirus 3CL^pro^ proteases flanked by autocatalytic sites were cloned into the P protein of a red fluorescent protein (dsRed) expressing VSV. The P protein with the internal 3CL^pro^ is functional and essential for viral genome replication and dsRed expression. In the absence of protease inhibitor, the P:3CL^pro^ protein is autocatalytically cleaved and dsRed is not expressed. In the presence of a protease inhibitor, the P protein is functional, the VSV genome replicates and dsRed is expressed. Using this system, we found that MHV 3CL^pro^ showed a weaker response to nirmatrelvir than to SARS-CoV-2 3CL^pro^ (**Fig. 1****A**). The sequence identity of the two proteins is only 50% (**fig. S2**), whereas the structures of the SARS-CoV-2 and MHV 3CL^pro^ enzymes are strongly conserved. However, the interaction site of nirmatrelvir (a distance of 5 Å or less from the compound) shows seven amino acid differences between the two enzymes, namely H164 - Q, M165 - L, P168 - S, V186 - R, R188 - A, T190 - V and A191 - V (counting from the first residue (serine) after the glutamine of the N-terminal cleavage site). We therefore suggest that MHV 3CL^pro^ is not an optimal proxy to study SARS resistance mutations.

### A VSV-based non-gain-of-function system was generated to predict SARS-CoV-2 3CL^pro^ mutations

To generate a safer alternative to selection of drug resistant SARS-CoV-2 for studying mutants, we engineered a chimeric VSV variant, where the intergenic region between the glycoprotein (G) and the polymerase (L) was replaced by the 3CL^pro^ of SARS-CoV-2 (**fig. S3A**). Upon translation, G, 3CLpro, and L form a surrogate polyprotein, which must be processed by 3CL^pro^ to generate the functional viral proteins G and L. This surrogate polyprotein mimics the polyprotein that is produced by SARS-CoV-2 as dimerization of 3CL^pro^ is obligate for its function (*18*, *19*) and cleavage of the cognate 3CL^pro^ N- and C-terminal motifs must occur for successful VSV replication. By applying an appropriate protease inhibitor (+PI), this processing is disturbed and therefore viral replication cannot occur (**fig. S3B**). Through passaging the chimeric VSV variant in presence of suboptimal concentrations of a protease inhibitor, 3CL^pro^ mutations that are generated by the error-prone viral polymerase (*20*, *21*) are selected for resistance to the inhibitor (**fig. S3C**).

In a first proof-of-concept study, we selected a mutant against the inhibitor GC376, which acquired the amino acid change in the 3CL^pro^ from phenylalanine to leucine at position 305 (F305L) in the autocatalytic cleavage motif at the C terminus of the protease. This virus gained a mildly faster replication kinetic and produced higher titers in the presence of GC376 and nirmatrelvir compared to the parental virus (**Fig. 1****B and C**). Related coronaviruses have leucine at position 305 as a preferred cleavage motif (**Fig. 1****D**); therefore the likely mechanism of the selection of F305L is autocleavage site optimization. We used the wild-type VSV-3CL^pro^ for subsequent mutation selection studies with nirmatrelvir. We also included the F305L as parental virus for further selection experiments, because the F305L mutation has been found in regional outbreaks (mostly in England) and has been deposited in the global initiative on sharing avian flu data (GISAID) database (*22*–*24*) with three different codon usages to obtain leucine instead of phenylalanine (**fig. S4A**). The mutants were variants of Delta, mainly the sublineage AY.4 (**fig. S4B**). We therefore assumed F305L could be an advantageous mutation that, in combination with further mutations, may give rise to protease inhibitor resistant lineages.

### Nirmatrelvir resistant 3CL^pro^ mutants were selected for in the VSV-3CL^pro^ system

**Fig. 2.**
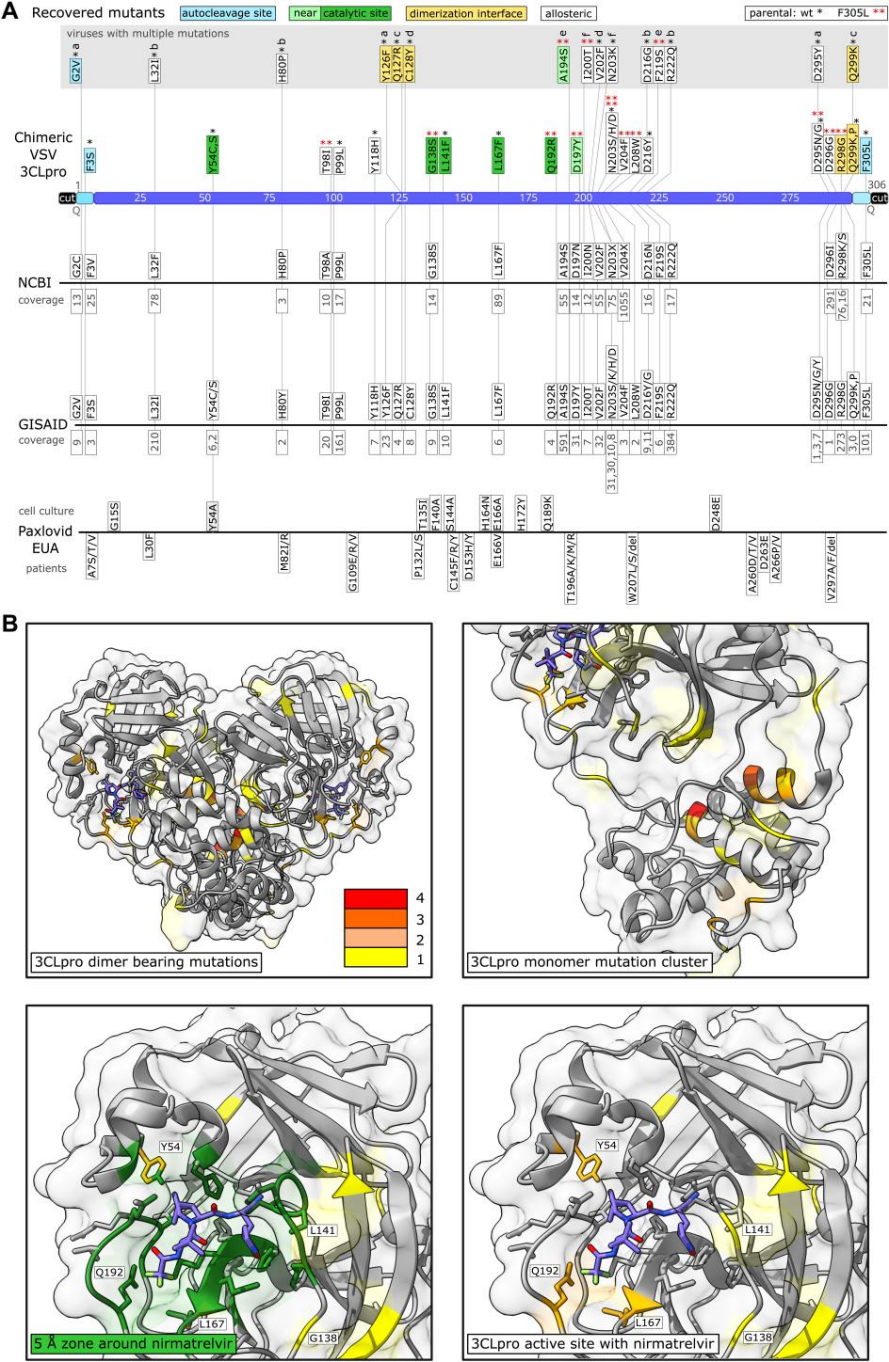
Sequencing of 3CL^pro^ escape mutants and comparison to data bases and Paxlovid EUA information. **(A)** Mutants were recovered from VSV-G-3CL^pro^-L wild-type (*) and the F305L variant (red **). Autocleavage site mutants are colored in turquoise, catalytic site mutants in green, near catalytic site mutants in light green, dimerization interface mutants in yellow and “allosteric” mutants in white. Viruses with more than one mutation are displayed above in a gray box and named a to f. The number of mutated sequences in the databases from NCBI and GISAID are displayed below the mutations in gray. If specific mutations were not present in the database, the residue is displayed with any mutation that occurred at this position. Multiple such different amino acid changes that were not selected in our virus are displayed with X (N203X, V204X). Mutations from the Paxlovid EUA are divided into mutations found in cell culture and mutations sequenced from treated patients. The coverage of mutation entries was obtained on June second, 2022. **(B)** Visualizations of mutation-affected residues are shown. Residues that were mutated one time are highlighted in yellow, two times in light orange, three times dark orange, and four times in red. The 3CL^pro^ protease dimer with bound nirmatrelvir (blue) was visualized in ChimeraX from the Protein Data Bank structure 7vh8 (*32*). Catalytic center mutations are within a range of 5 Å as visualized in dark green.

We next used the wild-type and F305L mutant viruses to select for nirmatrelvir resistant 3CLpro. BHK-21 cells in a 96-well plate were infected at a low multiplicity of infection (MOI; 0.01). Where cytopathic effects were visible in the first passage (25 out of 48 wells from parental wild-type and 17 out of 48 wells from parental F305L), supernatants were used for passaging individual wells with increasing concentrations (wild-type initial infection: 30 μM, second round: 40 μM, and third round: 50 μM; F305L initial infection: 50 μM, second round: 75 μM, and third round: 100 μM) of nirmatrelvir. At every passage, where cytopathic effects were observed again, supernatants were collected from the cell culture of individual 96-wells and transferred to individual new wells of a 96-well plate. At every passage, each well was sequenced individually, the target region being 3CL^pro^ and adjacent parts of G and L. We only counted mutants from unambiguous chromatogram peaks (as exemplified in **fig. S3C**). If, in the first or second passage there were still overlapping peaks, we sequenced the well again after the next passage. By this continuous selection pressure, the fittest mutant virus variant became dominant over the wild-type (and potential other mutants) in each well and made up the entirety of the genomic RNA, cDNA, and subsequent polymerase chain reaction (PCR) fragment. To finally exclude minority mutant populations that were not visible in a Sanger sequencing chromatogram, but could contribute to the resistance phenotype, mutations were later re-introduced into 3CL^pro^ measurement systems individually. We found 39 distinct mutations within 3CL^pro^ by Sanger sequencing. Viruses carried from one dominant mutation up to four mutations. The mutations were distributed over the entire sequence of 3CL^pro^ (**Fig. 2****A, table S1**). We categorized them into catalytic site, near-catalytic site, dimerization interface and autocleavage site mutants. A fourth category for all mutations not fitting the first three was chosen as “allosteric” mutants. The mutants Y54C, L141F, L167F and Q192R occurred in residues in very close proximity (within 5 Å of PDB 3CL^pro^ structure 7vh8, **Fig. 2****B**, **table S2**) to nirmatrelvir. We searched for the mutants in the National Center for Biotechnology Information (NCBI) Virus data base (*25*) and GISAID EpiCoV (*22*–*24*), and found most of the mutations, or at least the same residue with a different mutation, in deposited sequences with varying coverage (**Fig. 2****A, table S1**). We further subdivided GISAID entries into depositions made before and after the emergency use authorization of Paxlovid (nirmatrelvir/ritonavir) on 22th December, 2021 (**table S3**). An update of the Paxlovid EUA (18th March, 2022) included 3CL^pro^ mutants that were retrieved from patients treated with Paxlovid (nirmatrelvir/ritonavir) (**Fig. 2****A**). The update stated that it was unclear whether these mutations had clinical relevance (*26*).

### Replications kinetics and dose response were analyzed for selected 3CL^pro^ mutants

**Fig. 3.**
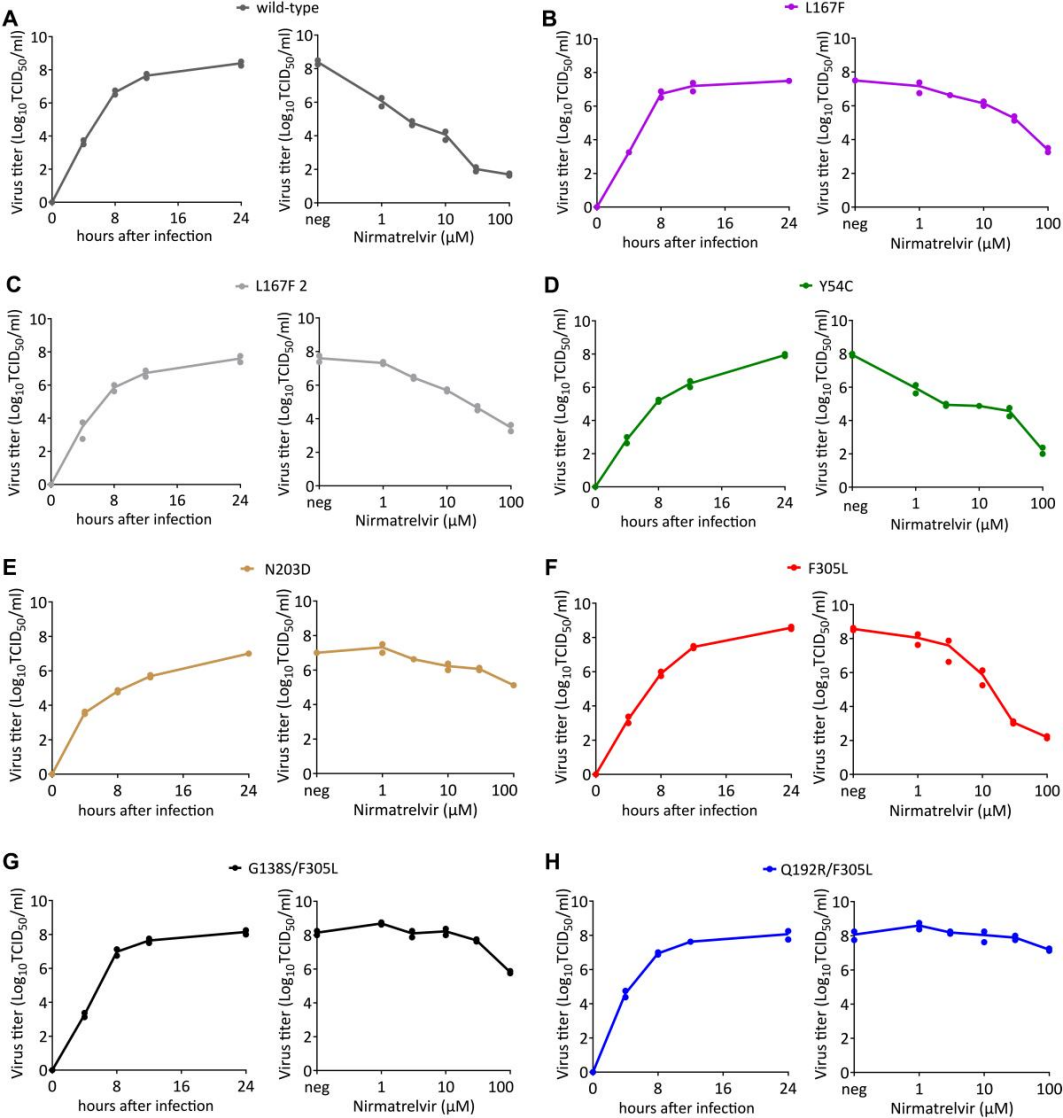
Replication kinetics and nirmatrelvir dose responses of parental VSV-G-3CL^pro^-L and mutant variants. **(A to H)** Replication kinetics and dose responses are shown for wild-type (**A**), L167F (**B**), L167F-2 (**C**), Y54C (**D**), N203D (**E**), F305L (**F**), G138S/F305L (**G**) and Q192R/F305L (**H**) VSV-G-3CL^pro^-L. Supernatants for replication kinetics were collected at indicated time points. Supernatants for virus nirmatrelvir dose response were collected 24 hours after infection. (n = 2 biologically independent replicates per condition with individual data points shown and connecting lines of mean values). neg, without nirmatrelvir; TCID_50_, 50% tissue culture infective dose.

To confirm these potential resistance mutations, we chose six virus samples to perform replication kinetics and dose response experiments. Mutants were selected for further testing based on two criteria. First, we chose virus variants with catalytic site mutations because alterations in drug binding residues (direct or indirect) are more likely to alter efficacy. Second, we chose the most frequently recovered mutant outside of the catalytic site. Four samples were derived from wild-type VSV-G-3CL^pro^-L and two were derived from the F305L variant. Supernatants for the replication kinetic experiments were collected at indicated time points after infection, and supernatants for the virus nirmatrelvir dose response experiments were collected 24 hours after infection. The replication kinetics revealed that all variants were still capable of replicating to high titers, suggesting that resistance mutations did not result in a strong negative effect on 3CL^pro^ activity (**Fig. 3**). The dose responses showed that wild-type VSV-G-3CL^pro^-L replication was inhibited by 10^6^-fold at 100 μM of nirmatrelvir, with a half maximal inhibitor concentration (IC_50_) of about 185 nM (**Fig. 3****A**). We tested two L167F variants, because this mutant arose twice independently. The similarity of their dose responses (**Fig. 3****B and C),** as well as the low variation of the biological replicates, suggests that the differences in the degree of resistance we observed between the mutants were not artifacts. We tested additional single mutants, namely the catalytic site mutant Y54C (**Fig. 3****D**), a mutant from the mutation cluster shown in **Fig. 2****B**, N203D (**Fig. 3****E**) and the autocleavage site mutant F305L (**Fig. 3****F**). To test if mutants that were selected from the F305L background had increased resistance, we also tested double mutants G138S/F305L (**Fig. 3****G**) and Q192R/F305L (**Fig. 3****H**). We observed the strongest resistance phenotype in the double mutant Q192R/F305L, replicating to high viral titers with a pronounced cytopathic effect (**fig. S5**) even in the presence of 100 μM nirmatrelvir.

### Re-introduction of 3CL^pro^ mutations confirms their resistance phenotype

**Fig. 4.**
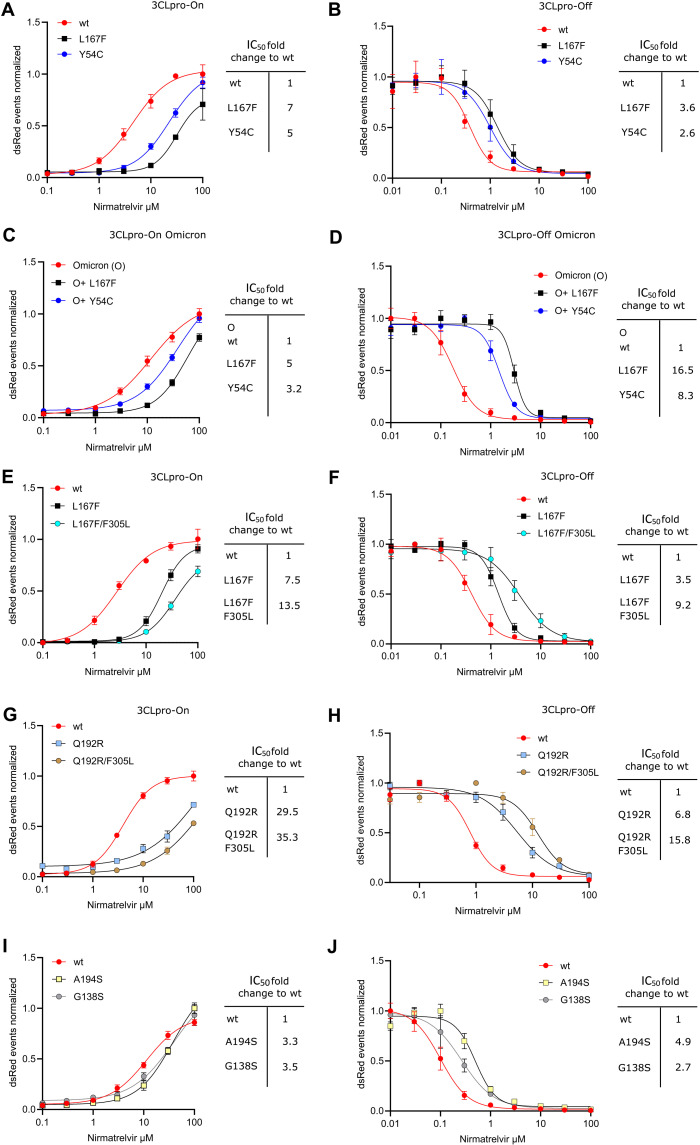
Re-introduction of individual or dual 3CL^pro^ mutations confirms their resistance phenotype. A graphic representation of the 3CL^pro^-on and 3CL^pro^-off system used to measure the inhibitory activity of the protease inhibitor against the different 3CL^pro^ mutants can be found in **fig S3**. **(A)** Gain-of-signal assay results are shown for single catalytic site mutations Y54C and L167F with nirmatrelvir. Data are presented as the standard deviation (SD) of n = 3 biologically independent replicates per condition. **(B)** Loss-of-signal assay results are shown for single catalytic site mutations Y54C and L167F with nirmatrelvir. Data are presented as the SD of n = 4 biologically independent replicates per condition. **(C)** Gain-of-signal assay results are shown for catalytic site mutations Y54C and L167F in combination with the Omicron 3CL^pro^ signature mutation P132H. Data are presented as the SD of n = 4 biologically independent replicates per condition. **(D)** Loss-of-signal assay results are shown for single catalytic site mutations Y54C and L167F in combination with the Omicron 3CL^pro^ signature mutation P132H. Data are presented as the SD of n = 4 biologically independent replicates per condition. **(E)** Gain-of-signal assay results are shown for double mutant L167F/F305L versus wild-type and single mutant L167F. Data are presented as the SD of n = 4 biologically independent replicates per condition. **(F)** Loss-of-signal assay results are shown for double mutant L167F/F305L versus wild-type and single mutant L167F. Data are presented as the SD of n = 4 biologically independent replicates per condition. **(G)** Gain-of-signal assay results are shown for double mutant Q192R-F305L versus wild-type and single mutant Q192R. Data are presented as the SD of n = 4 biologically independent replicates per condition. **(H)** Loss-of-signal assay results are shown for double mutant Q192R/F305L versus wild-type and single mutant Q192R. Data are presented as the SD of n = 4 biologically independent replicates per condition. **(I)** Gain-of-signal assay results are shown for mutants A194S and G138S versus wild-type. Data are presented as the SD of n = 4 biologically independent replicates per condition. **(J)** Loss-of-signal assay results are shown for of mutants A194S and G138S versus wild-type. Data are presented as the SD of n = 4 biologically independent replicates per condition.

As shown in **table S1**, VSV-induced 3CL^pro^ mutations were observed after the first passage when nirmatrelvir was applied. To validate the resistance data of replication-competent VSV-3CL^pro^ and at the same time exclude the effects of potential additional mutations arising within the dose response experiment, we re-introduced some of the catalytic center mutations (Y54C, L167F, Q192R) into a recently developed protease activity measurement tool based on replication-incompetent VSV (*17*) (**fig. S1A and B**). In brief, the protease activity measurement tools comprise replication-incompetent VSV-dsRed variants missing either the viral phosphoprotein (ΔP) or polymerase (ΔL). These viruses are complemented with either an INTRAmolecular-3CL^pro^ tagged phosphoprotein or and INTERmolecular GFP-3CL^pro^-L fusion protein, respectively. The P:3CL^pro^ or GFP-3CL^pro^-L proteins are expressed in cells from transfected plasmids. The cells are then infected with the replication incompetent VSV-dsRed variant and treated with inhibitors. An intramolecular 3CL^pro^ tag in combination with VSV-ΔP-dsRed constitutes a gain-of-signal or “on”-assay. An intermolecular 3CL^pro^ tag in combination with VSV-ΔL-dsRed constitutes a loss-of-signal or “off”-assay. We found that the identified single catalytic center mutations indeed conferred partial resistance against nirmatrelvir of the 3CL^pro^ from the Wuhan-1 as well as the Omicron SARS-CoV-2 variant (Omicron signature mutation in 3CL^pro^ P132H) (**Fig. 4****A to D**), which could be further enhanced by introduction of a second mutation in the autocleavage site (F305L, **Fig. 4****E to H**). The mutation Q192R arose in the F305L parental virus. Introducing Q192R alone reduced 3CL^pro^ activity mildly, as we observed by increased values in 3CL^pro^-On-Q192R at low nirmatrelvir concentrations. Adding F305L as second mutation, thereby restoring the original combination from the double mutant virus, rescued this phenotype (**Fig. 4****G**). A randomly selected combination of catalytic center mutations led to a strong loss in enzymatic activity (**fig. S6A and B**). We further introduced two mutants (A194S, G138S) into the 3CL^pro^ measurement assays, which also conferred resistance to nirmatrelvir (**Fig. 4****I and J**).

### Nirmatrelvir and GC376 react differently to 3CL^pro^ mutants

**Fig. 5.**
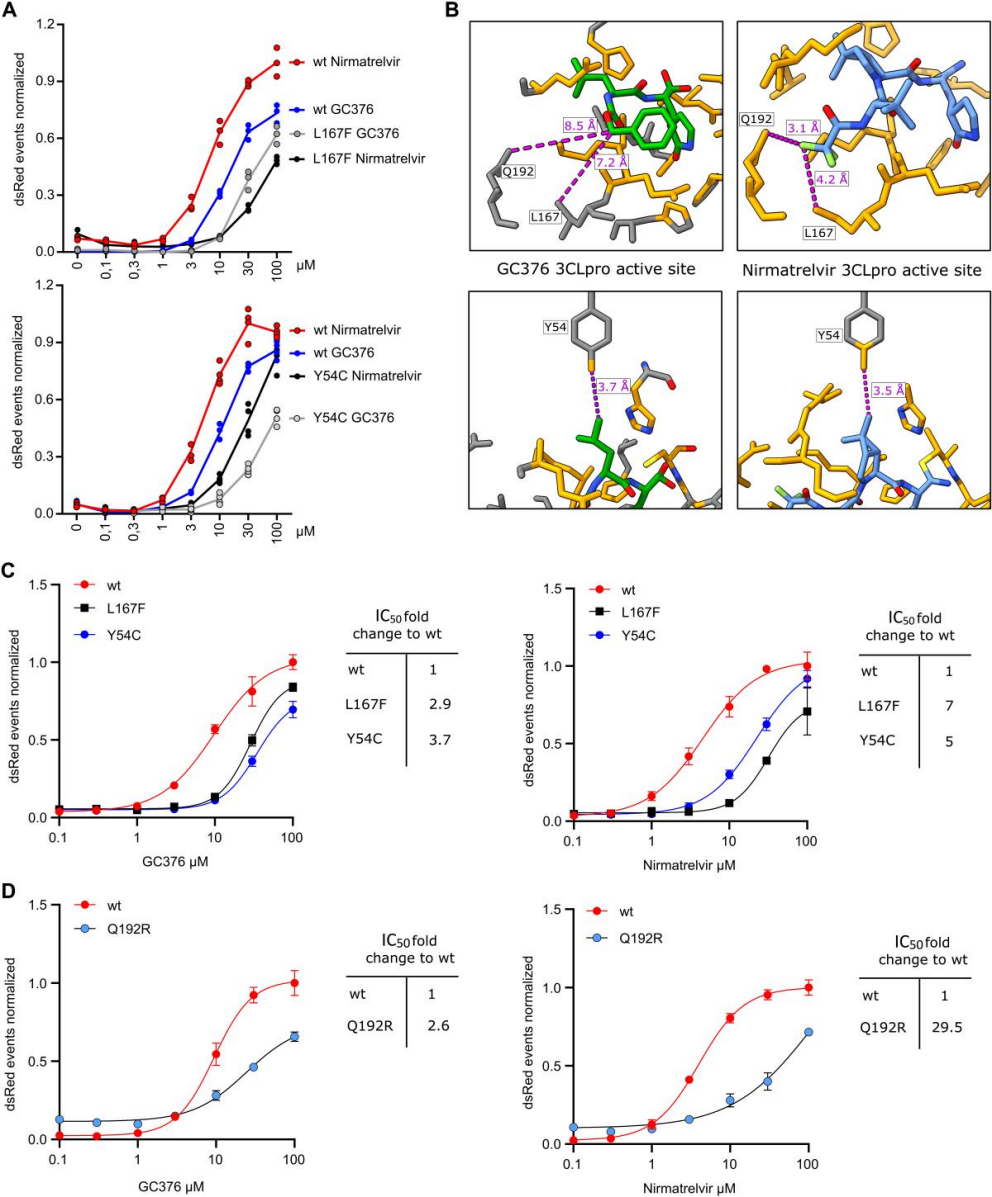
Nirmatrelvir and GC376 react differently to mutants. **(A)** Gain-of-signal assay results are shown for single mutants Y54C and L167F versus wild-type tested with GC376 and nirmatrelvir (Y54C: n = 4, L167F: n = 3 biologically independent replicates per condition). **(B)** GC376 (PDB: 7k0g) and nirmatrelvir (PDB: 7vh8) 3CL^pro^ crystal structures are shown with GC376 in green (and colored by heteroatom) and nirmatrelvir in light blue (and colored by heteroatom) and proximal residues in orange (within zone of 5 Å). Compound to residue distances are shown with dotted purple lines. **(C)** Fitting of gain-of-signal assay results are shown for single mutants Y54C and L167F versus wild-type tested with GC376 and nirmatrelvir. **(D)** Fitting of gain-of-signal assay results are shown for single mutant Q192R versus wild-type tested with GC376 and nirmatrelvir. Data in (C and D) are presented as the SD of n = 4 biologically independent replicates per condition.

Comparing GC376 to nirmatrelvir in the 3CL^pro^ Y54C and L167F mutants directly revealed that these mutants react differently to the compounds. Y54C confers a similar resistance to GC376 as to nirmatrelvir (**Fig. 5****A**). GC376 and nirmatrelvir interact similarly with the residue Y54, whereas L167 and Q192 are distant to GC376 and close to nirmatrelvir (within 5 Å) (**Fig. 5****B**). L167F and Q192R appeared to affect the activity of GC376 less than nirmatrelvir (**Fig. 5****C and D**, **tables S4 and S5**). Nirmatrelvir IC_50_ values were especially high in the 3CL^pro^-On construct. We sought to improve the assays sensitivity by changing the read-out method from a FluoroSpot to a flow cytometry-based readout. With this approach, we could decrease the IC_50_ of the wild-type 3CL^pro^-On to 0.91 μM of nirmatrelvir (**fig. S6C, table S6**).

### Confirmation of resistance mutations in a second cell-based assay system, biochemical assay, and with recombinant SARS-CoV-2

**Fig. 6.**
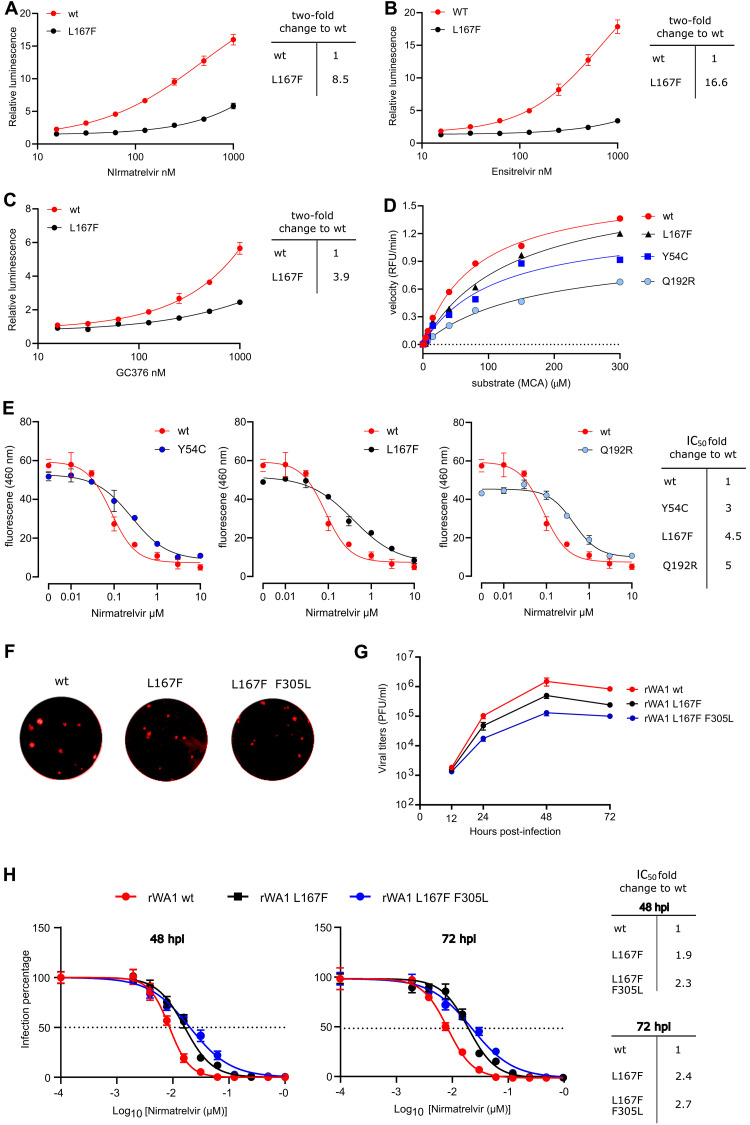
Cross-testing mutants and validation of enzyme kinetics. **(A to C)** Cross validation with cellular gain-of-signal assay based on Src-3CL^pro^-Tat-Luc polyprotein. Wt Src-3CL^pro^-Tat-Luc and L167F mutant were tested with nirmatrelvir **(A),** ensitrelvir **(B),** and GC376 **(C)**. Data are presented as mean ± the standard deviation (SD) of n = 3 biologically independent replicates per condition. **(D)** Enzyme kinetics were measured for wt 3CL^pro^ and mutants with the substrate Ac-Abu-Tle-Leu-Gln↓MCA releasing the fluorogenic molecule MCA. Two biologically independent replicates per condition were used to calculate means for slopes. Slopes were used to compose a Michaelis Menten graph. Relative fluorescent units / minute (RFU/min) were used to plot the velocity of enzymes in the Michaelis Menten graph. **(E)** Results of a biochemical assay used for cross-validation are shown. In presence of an appropriate protease inhibitor, 3CL^pro^ cannot cleave the substrate and fluorescence is low. Without inhibitor, 3CL^pro^ cleaves the substrate peptide (KTSAVLQSGFRKME), quencher (DABCYL) and fluorogen (EDANS) are separated, and fluorescence increases. Nirmatrelvir dose responses are shown for wt versus mutant 3CL^pro^ variants; IC_50_ fold changes show varying resistance of the mutant enzymes. Data are presented as mean ± the standard deviation (SD) of n = 2 biologically independent replicates per condition. **(F)** Recombinant wt, L167F and L167F/F305L SARS-CoV-2-mCherry (rWA1) exemplary plaques were imaged without magnification. **(G)** Replication kinetics are shown for recombinant wt SARS-CoV-2 (rWA1) versus L167F single and L167F F305L double mutant viruses. Data are presented as mean ± the standard deviation (SD) of n = 3 biologically independent replicates per condition. **(H)** Results are shown comparing the resistance of L167F single and L167F F305L double mutants to nirmatrelvir versus wt rWA1 expressing mCherry (top). The fold change in IC_50_ values are shown on the right. Data are shown for 48 and 72 hours post infection (hpi). The dotted line indicates 50% inhibition. Data are presented as mean ± SEM from quadruplicate wells of 2 independent experiments.

The resistance phenotype of L167F observed with gain- and loss-of-signal assays was confirmed using another recently published cellular system (*27*). In this complementary assay, a polyprotein of Src, 3CL^pro^ with N-and C-terminal autocleavage sites, HIV Tat, and luciferase was used to repress transcription when 3CL^pro^ was active (**fig. S7**). Bona fide chemical inhibitors blocked 3CL^pro^ activity and restored luciferase signal in a dose-dependent manner (**Fig. 6****A to C**). Similar to the results described above, L167F was more resistant to nirmatrelvir than GC376 (**Fig. 6****A and C, table S7**). Furthermore, this mutant was most resistant to ensitrelvir, a recently developed compound in clinical trials in both the Src-3CL^pro^-Tat-Luc (**Fig. 6****B, table S7**) (*28*) and our assay (**fig. S8, table S8**).

For further confirmation of resistance phenotypes, we purified recombinant enzymes (**fig. S9A to C**). We tested catalytic activity with a substrate dose-response kinetic experiment with purified wild-type 3CL^pro^ versus mutants Y54C, L167F, and Q192R with the substrate Ac-Abu-Tle-Leu-Gln↓MCA releasing the fluorogenic molecule 7-amino-4-methylcoumarin (AMC) (**fig. S10A**). The ratio between the catalytic constant or turnover rate (k_cat_) and the Michaelis-Menten constant (K_m_), displayed as k_cat_/K_m_, showed that some of the mutants partially lose catalytic activity, most notable Q192R, which was in line with the cellular assays (**Fig. 6****D, table S9**). For further resistance confirmation we applied a biochemical fluorescence resonance energy transfer (FRET) assay, which uses a quencher (DABCYL) and a fluorogenic substance (EDANS), which are connected by a peptide (KTSAVLQSGFRKME) that is recognized and cleaved by 3CL^pro^ (**fig. S10B**). Upon cleavage, fluorescence of EDANS increases. All three mutant 3CL^pro^ enzymes were more resistant to nirmatrelvir than the wild-type 3CL^pro^ (**Fig. 6****E, table S10**).

Lastly, we confirmed our findings in recombinant SARS-CoV-2 viruses expressing a reporter gene (**fig. S10C**) (*29*, *30*). The recombinant SARS-CoV-2 variant expressing mCherry used for mutagenesis, aside from its transgene, was sequence identic to the Wuhan-1 variant (*29*, *30*) (**Data file S1**). Viruses carrying L167F alone and in combination with F305L were able to replicate, but replicated slower than wt virus and produced smaller plaques (**Fig. 6****F and G**). The mutations introduced into recombinant SARS-CoV-2-mCherry had been found in clinical samples prior to our study (**Data file S2**). As expected, both L167F single and L167F/F305L double mutants were more resistant to nirmatrelvir than the wild-type (**Fig. 6****H, table S11**).

### Structural modelling of mutant 3CL^pro^ variants

To explore potential mechanisms of resistance, we performed molecular modelling with an in silico alanine mutation scanning as well as resistance mutation scanning with Molecular Operating Environment (MOE) suite (*16*) and the Robetta service (*15*). MOE modelling was based on the PDB structure 7rfw (*31*), and Robetta modelling was based on 7vh8 (*32*), both of which are 3CL^pro^ structures with high nirmatrelvir occupancy. Experimental alanine scanning (*33*, *34*) as well as in silico alanine screening (*35*, *36*) is routinely utilized to evaluate the impact of single amino acid mutations on protein structure, and the models can provide plausible explanations for the structural basis of nirmatrelvir resistance.

**Fig. 7.**
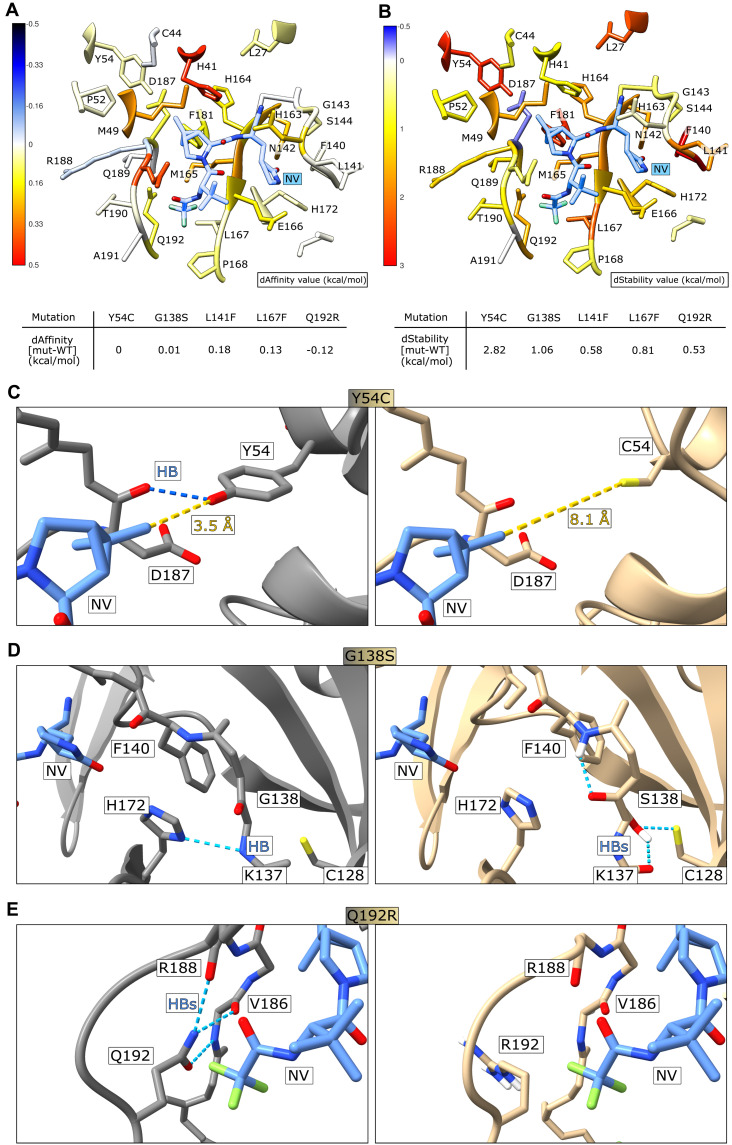
Structural modelling of mutant 3CL^pro^ variants. **(A)** Colorimetric mapping of the dAffinity value (kcal/mol) by virtual alanine scanning with MOE suite. Residues within 5 Å of the nirmatrelvir position are displayed. Colors range from blue (negative values, indicating increased protein-ligand affinity) to red (positive values, indicating decreased protein-ligand affinity). The nirmatrelvir (NV) structure is shown in light blue. **(B)** Colorimetric mapping of the dStability value (kcal/mol), computed as above for (A). Colors range from blue (negative values, indicating increased in the protein stability) to red (positive values, indicating decreased protein stability. **(C)** The catalytic center of 3CL^pro^ from PDB structure 7vh8 is shown with nirmatrelvir bound. Y54 (left) forms a strong hydrogen bond (HB, highlighted with a blue dashed line) with D187, whereas nirmatrelvir is at a distance of 3.5 Å (yellow dashed line). The exchange of Y54 with C (right) leads to a loss of the hydrogen bond to D187 and makes room in the nirmatrelvir binding pocket due to the smaller side-chain of cysteine versus tyrosine. **(D)** G138 (left) contacts H172 with a hydrogen bond. S138 (right) forms several new hydrogen bonds with the backbone hydrogen of F140, backbone oxygen of K137 and the sulfur of C128. **(E)** Q192 (left) forms hydrogen bonds with the oxygen and nitrogen of V186, the oxygen of R188, and stabilizes the polar contact to the CF_3_ group of nirmatrelvir. R192 (right) disrupts this hydrogen bond network; subsequent rearrangement could form additional interactions with the CF_3_ group.

**Fig. 7****A** shows that the most important losses of binding affinity are primarily related to mutation of residues whose sidechains directly contact the ligand, such as H41, M49, N142, H163, M165 and Q189, and secondarily to other residues lining the binding site like Y54, H164, E166, P168, D187, and Q192. Residues with hydrophobic sidechains, such as L27, Y54, F140, L167 and F181 seem to have a pivotal role in the structural integrity of the binding site (**Fig. 7****B**), despite having a negligible impact on the variation of binding affinity.

The primary effect of Y54C in these models is the disruption of a stabilizing inter-protein hydrogen bond to the backbone oxygen of D187 and disruption of additional weak but stabilizing interactions with surrounding hydrophobic residues such as a π-charge interaction between the phenyl ring of Y54 and the guanidinium group of R40. No major direct interaction to the 3.5 Å distant C20 methyl group of nirmatrelvir exists (**Fig. 7****C, fig. S11A**). However, loss of the critical hydrogen bond between loop region 43 to 55 and the adjacent loop around D187 allows for a structural rearrangement destabilizing the distal part of the binding site, likely increasing the inherent plasticity of this protein region.

Residue G138 lies in a solvent accessible loop, with backbone torsion angles in the β sheet region. Replacing it with a polar serine (G138S) while maintaining the same backbone conformation led to the Cβ of S138 pointing into the protein interior, and all of the preferred rotamers led to unfavorable interactions or required a rearrangement of the affected region. Formation of new hydrogen bonds, for example with a backbone hydrogen of F140 and the sulfur of C128 (**Fig. 7****D**) likely led to a rearrangement of the S1 subpocket, which is responsible for hosting the terminal carboxamide moiety that mimics the P1 glutamine in natural peptide substrates.

Supervised Molecular Dynamics simulations of the nirmatrelvir-3CL^pro^ recognition process revealed how L141 is one of the first residues that is contacted during the approach of nirmatrelvir into the binding site (*37*). In the L167F mutant, the larger sidechain of phenylalanine cannot be accommodated without a structural rearrangement, which likely leads to repulsive interaction between the trifluoromethyl (CF_3_) moiety of nirmatrelvir and weakening its interactions with other proximal residues such as N142, which are thought to play a pivotal role in maneuvering the ligand entrance in the catalytic pocket (*37*). As anticipated in the alanine scan, the L167F mutation seems to have an indirect effect on the binding affinity by alteration of the β sheet that constitutes the lower portion of the binding site, where a set of hydrogen bonds are established between nirmatrelvir and the backbone of both H164 and E166 (**fig. S11B**). A similar distortion of the binding pocket by the bulkier phenylalanine has also been described recently (*10*).

Finally, polar Q192 stabilizes a solvent exposed loop participating in hydrogen bonds to backbone oxygen and nitrogen of V186, backbone oxygen of R188, and a stabilizing contact to the CF_3_ group of nirmatrelvir. Replacement with positively charged R192 disrupted this network, which likely results in a structural rearrangement and altered binding to nirmatrelvir (**Fig. 7****E**). The slight increase in binding affinity of Q192R concurrent with protease destabilization (**Fig. 7****A and B**) could be explained by recontouring of the subpocket hosting the negatively polarized CF_3_ moiety of nirmatrelvir and interacting with positively charged R192 (**Fig. 7****E, fig. S11C**). Despite the predicted, marginally more favorable interaction with nirmatrelvir, an overall unfavorable effect of the mutation could still be possible due to altered sequestration of nirmatrelvir and the destabilization of the loop region lining the S2 and S4 subpocket of the catalytic site, where important residues such as Q189 are located.

## DISCUSSION

In our study, we selected mutations in the main protease 3CL^pro^ of SARS-CoV-2 against the protease inhibitor nirmatrelvir with a non-gain-of-function system based on VSV. The selected mutations were confirmed in two cellular assays and in one biochemical assay, along with confirmation using recombinant SARS-CoV-2. For the catalytic site mutations, a resistance mechanism was postulated based on mapping the mutations onto the co-crystal structure of 3CL^pro^-nirmatrelvir and generating mutant models with Robetta (*38*).

In previous initial resistance studies leading to emergency use authorization of Paxlovid (nirmatrelvir/ritonavir), the 3CL^pro^ of a related coronavirus, MHV, was used to select for resistance mutations. The 3CL^pro^ of SARS-CoV-2 and MHV share 50% sequence identity. In this study, we compared the activity of 3CL^pro^ of SARS-CoV-2 and MHV and found that MHV 3CL^pro^ responded only mildly to nirmatrelvir in our gain-of-signal assay (*17*). Although the structures of SARS-CoV-2 and MHV 3CL^pro^ are conserved, we propose that the low amino acid sequence identity alters the binding pocket affinity to nirmatrelvir sufficiently to reduce the sensitivity against the inhibitor. Key corresponding residues of the binding pocket (within 5 Å or less) are different, namely H164 - Q, M165 - L, P168 - S, V186 - R, R188 - A, T190 - V and A191 - V. Furthermore, amino acid changes that occurred in our selection experiments, Y126F and F305L are already present in the MHV 3CL^pro^ sequence. Taken together, we argue that MHV 3CL^pro^ was not an optimal proxy for resistance studies.

Recently, chimeric VSV variants with SARS-CoV-2 spike were used to predict spike protein immune escape mutations by selecting against neutralizing serum (*39*–*41*). The fast occurrence of mutations was facilitated in those studies by the high error rate of the VSV polymerase (*20*, *21*). In a similar approach, we exploited this high error rate in a recombinant VSV expressing 3CL^pro^ to select for 3CL^pro^ mutations that confer resistance against protease inhibitors. The 3CL^pro^ was used to replace the function of an intergenic region between the viral glycoprotein (G) and the polymerase (L). The intergenic regions of VSV are responsible for separate gene expression, which in other viruses is accomplished by a polyprotein and proteases. Although this polyprotein of VSV-G-3CL^pro^-L is only a surrogate to the one in SARS-CoV-2, the cognate cleavage sites, the requirement for dimerization of the protease for proper function (*18*, *19*) and the context of a replicating virus in the cell make this approach an attractive proxy.

Initially, we selected the 3CL^pro^ mutant F305L using GC376, which showed reduced sensitivity to GC376, as well as to nirmatrelvir. This mutation lies in the 3CL^pro^ cleavage site that flanks 3CL^pro^ at its C terminus. Interestingly, the LQ motif found in the mutated site is indeed known to be preferred over FQ as a target motif for 3CL^pro^ (*42*–*44*), which may explain the reduced sensitivity of the F305L mutant to the protease inhibitors. We then selected both wild-type and the F305L mutant against nirmatrelvir. We also used F305L as parental virus because we found that this variant existed already in regional outbreaks (mainly in England), underlining the viability of this mutation and its potential replicative advantage. These clusters were mainly of the Delta subvariant AY.4. Delta was replaced gradually by Omicron, which may have ended the spread of the Delta F305L. Nevertheless, we also found combinations of the Omicron signature mutation P132H with F305L. We were therefore interested in finding potential combinations of F305L with further protease inhibitor resistance mutations, assuming that the combination would show a higher degree of resistance than single mutations, which we did observe. Mutations from both wild-type and F305L were selected that ultimately allow the mutants to escape the inhibitor. Resistance phenotypes were confirmed by dose response experiments and re-introduction of mutations into recently developed protease activity measurement systems (*17*) as well as alternative methods such as biochemical (*45*) and cellular assays (*27*).

We collected a total of 39 unique mutations, of which Y54C, L167F, N203D and D216Y occurred twice independently. F305L was selected for with both GC376 and nirmatrelvir. Six out of 39 occurred in the catalytic site, two near the catalytic site, seven at the dimer interface, three in the autocleavage sites and 21 in the rest of the 3CL^pro^ sequence (which we called “allosteric” mutations). First, we confirmed catalytic site mutants (Y54C, G138S, L167F and Q192R), where the resistance mechanism is likely straight forward: the steric disturbance of nirmatrelvir binding. Then, we tested the near-catalytic site substitution A194S, which is more prevalent than the previous catalytic site mutants in virus isolates. In GISAID, this particular mutation can be found in over 800 sequence depositions in the variants of concern Alpha, Gamma, Delta, Lambda, and Omicron. Changes of the residue A194 in general are frequent with over 3000 entries. Although it is not known if this mutant was selected for by the use of nirmatrelvir in patients, the fact that it is a resistant mutant and prevalent in virus sequences makes is a variant worth tracking. We further combined L167F and Q192R with the autocleavage site mutation F305L, which further increased the resistance. The combination of Y54C and L167F with the Omicron signature mutation P132H also conferred increased resistance, highlighting the potential relevance of these mutations for the Omicron variant. The substitution F305L was described as resistance mutation in this study. An adjacent mutant, T304I, was found in nirmatrelvir selection experiments with authentic SARS-CoV-2 (*9*) and the suggested mechanism was autocleavage site optimization. Given F305L is also likely an autocleavage site optimizing mutant, we did not test it further in a biochemical assay, since such assays use mature protease in which autoprocessing does not play a role. Lacking an appropriate method, we therefore did not investigate the mechanism of F305L. Nevertheless, such mutants merit further study in assays that can elucidate the mechanism of action.

One technical particularity in the 3CL^pro^-On construct is that the nirmatrelvir IC_50_ values are higher than generally reported in the literature. However, IC_50_ values are generally higher in cell-based assays than in biochemical assays as we described previously (*17*). In brief, in the excess of an inhibitor and constant renewal of protease fusion protein, signals are expected to plateau later than in a biochemical assay with a fixed amount of enzyme. Furthermore, the screening method used in this study to assess mutants was originally developed as high-throughput screening tool for 3CL^pro^ inhibitors using a FluoroSpot reader that allows fast sampling (*17*). We improved the assays sensitivity by changing the read-out method from FluoroSpot to flow cytometry-based sampling. Flow cytometry sampling is more sensitive, but also more time consuming. Flow cytometry read-outs captured milder degrees of inhibition and resulted in a more gradual signal increase; therefore, this resulted in lower IC_50_ values in 3CL^pro^-On assay (0.91 μM of nirmatrelvir, which is closer to the published range of 74.5 (66.5 to 83.4) nM) (*46*).

We cross-validated several of our mutants in different assays. We confirmed the resistance data of L167F with a previously published cellular assay (*27*) and the mutants Y54C, L167F and Q192R with a biochemical assay (*47*). We showed also in a biochemical assay that the kinetic metrics of the mutants Y54C, L167F, and Q192R are attenuated to varying degrees. However, the VSV-chimeric viruses containing resistant 3CL^pro^ showed little fitness loss. In three recent preprints, L167F as well as various mutants at Q192 were identified to be resistance mutations in authentic SARS-CoV-2 (*9*, *10*, *12*).

Lastly, we confirmed the viability and resistance of the single mutant L167F and in combination with F305L in a previously published recombinant SARS-CoV-2 expressing a reporter gene (*29*, *30*), finally confirming the validity of our mutation prediction tool based on VSV as well as the resistance mutations identified. Genetically modifying highly pathogenic viruses such as SARS-CoV-2 can be considered as so-called gain-of-function experiments if the recombinant virus is more apt to cause disease, or if treatment is made more difficult than the wt variant. We therefore applied several safety measures such as using a virus for mutagenesis that was sequence identic to the Wuhan-1 variant. Therefore, compared to currently circulating viruses, it has not undergone extensive evolution and if set free, would unlikely be able to compete with current Omicron variants. Importantly, other previously described antivirals approved for use in humans have been shown to inhibit viral replication of this Wuhan-1 strain. Moreover, current vaccines used in humans to protect against SARS-CoV-2 have been developed based on the sequence of the Spike glycoprotein of this, or a similar, Wuhan-1 strain. Thus, neutralizing antibody responses induced by these vaccines will be able to protect against these recombinant viruses. Secondly, the plaques the mutant recombinant viruses formed in Vero E6 cells were found to be smaller than that of wild-type recombinant virus and replication kinetics also indicate mild attenuation. Third, the mutations introduced into recombinant SARS-CoV-2-mCherry had been found in clinical samples already.

In this study, we identified several mutations such as Y54C, G138S, L167F, Q192R, A194S and F305L in the SARS-CoV-2 3CL^pro^ that confer resistance to the 3CL^pro^ inhibitors nirmatrelvir and GC376. To understand these mutations in light of the Omicron variant, we combined two of our most intensively studied mutations, Y54C and L167F with the Omicron 3CL^pro^ signature mutation P132H. These results showed that the mutations are functional, thereby confirming their potential relevance in this context of the Omicron variant.

Complementary structure modelling approaches based on Robetta and the Molecular Operating Environment (MOE) reveal potential effects of the catalytic site mutants Y54C, G138S, L141F, L167F and Q192R. Y54C and G138S seem not to directly affect nirmatrelvir binding, but may lead to a restructuring of the catalytic site, thereby indirectly affecting the binding site. L141F is likely impacting the early sampling of the catalytic pocket by nirmatrelvir. L167F may distort the distal region of the binding pocket. Lastly, Q192R could strengthen a polar interaction with nirmatrelvir, which may alter sequestration of the compound in an unfavorable position concurrent with destabilization of a loop containing important nirmatrelvir-catalytic site interaction partners such as Q189.

Our study has limitations. The mutations generated in VSV occurred in an artificial polyprotein, which, like pp1a or pp1ab, comprises precursors for large protein subunits and requires 3CL^pro^ dimerization for autocleavage. Nevertheless, the polyprotein structure is different, which could result in mutations not relevant in authentic SARS-CoV-2. Along the same line, in this artificial polyprotein, only autocleavage or cis-cleavage occurs, whereas in SARS-CoV-2, the mature 3CL^pro^ additionally cleaves distant or trans-cleavage sites. It has been shown that autocleavage of 3CL^pro^ in coronaviruses is a stepwise process with distinct N- and C-terminal autocleavage binding pocket confirmations (*48*, *49*), where the C-terminal autocleavage occurs after N-terminal autocleavage and might resemble a matured structure as in the trans-cleavage confirmation. Even so, this system could, in theory, disregard trans-cleavage specific mutants, if such exist. Finally, we did not elucidate the exact mode of resistance of the different mutants described in this study. Although we modelled catalytic site mutations and describe a plausible mechanism for autocleavage site mutants, solving crystal structures was beyond the scope of this work and remains for future studies.

In conclusion, our findings argue for a highly selective application of protease inhibitors to patients at increased risk of severe disease, as extensive, unselective use is expected to rapidly lead to emergence of drug resistance. Furthermore, the combination of different drugs is a proven strategy to avoid resistance mutations, as has been shown for HIV (*3*) and HCV (*2*) therapy. As more compounds became available, combinations including classes of inhibitors targeting distinct viral functions, such as protease and polymerase inhibitors, may be an effective strategy. However, as we observed in this study, 3CL^pro^ mutants can react differently to specific compounds. Therefore, even the combination of different protease inhibitors could lower the risk of viral escape.

## MATERIALS AND METHODS

### Study design

The overall rationale of the study was to develop a mutation selection tool based on VSV and to describe mutants as proof-of-concept for that tool. The study was performed on cell lines and in-silico, and no animal husbandry or human participants were involved. Human and monkey cell lines with replicating BSL-1, 2 and − 3 viruses were treated with protease inhibitors to observe resistance phenotypes in appropriate facilities. Viral titers were determined using TCID_50_ and plaque assays. Measurement readouts were fluorescence-based, detected by flow cytometry, ELISpot and multi-well readers. Autofluorescent fibers were excluded automatically and manually from spot counting in the ELISpot readout. Experiments were neither blinded nor randomly distributed to experimenters. We chose sample sizes empirically based on experience from former studies. At least two and up to four biologically independent replicates were performed per condition. Biologically independent meant distinct wells with the same condition, not multiple measurement of the same wells (technical replicates). Resistance phenotypes were reproduced at least twice, usually more often and in different combinations (comparing single mutants to each other and the wild-type or wild-type to single and double mutants). Representative measurements were chosen to compile graphs and figures.

### Cloning strategies

The chimeric VSV variant with 3CL^pro^ instead of the intergenic region between G and L was cloned by Gibson assembly (New England Biolabs, NEB) (*50*). A VSV-G plasmid (*51*) was digested with KpnI and HpaI (NEB), removing a C-terminal part of G, the intergenic region and a small N-terminal part of L. Insert fragments were generated as follows. Missing C-terminal G with an additional overhang to the N-terminal cleavage site of 3CL^pro^ was amplified with primers 33n-before-KpnI-for and G-cut1-rev. 3CL^pro^ with its N- and C-terminal cleavage sites and a C-terminal overhang to L was amplified from Wuhan-1 (NCBI Reference Sequence: NC_045512.2) cDNA with primers cut1-for and cut2-L-rev. The N-terminal missing L sequence was amplified with primers cut2-L-for and 33n-after-HpaI-rev. For subsequent Gibson assembly, the fragments were ligated in a fusion PCR using the outer primers 33n-before-KpnI-for and 33n-after-HpaI-rev with all three fragments as templates. The cloning primers for VSV vectors are shown in **table S12 and** the annotated sequence is shown in **data file S3**.

3CL^pro^-Off and -On point mutants were generated by mutagenic Gibson assembly on parental plasmids (GenBank accession codes: 3CL^pro^-Off: 25684003; 3CL^pro^-On: 2568399). For 3CL^pro^-Off mutants, a lentiviral expression plasmid expressing VSV L (identical sequence as blasticidin 3CL^pro^-Off plasmid without GFP and 3CLpro) was digested with HpaI, which removed the cPPT/CTS and CMV promoter sequences and a small N-terminal part of L. This missing sequence was replaced with the identical sequence from 3CL^pro^-Off with the addition of the N-terminal 3CL^pro^ sequence up to the respective mutation site with primers blasticidin-for and 3CL^pro^-*mut-x*-rev, where *mut-x* is the mutation of interest. The C-terminal part of 3CL^pro^ and the small missing fragment of L were generated by PCRs on parental vectors with primers 3CL^pro^-*mut-x*-for and 33n-after-HpaI-rev.

For 3CL^pro^-On mutants, a lentiviral hygromycin vector (modified from Addgene pLenti CMVie-IRES-BlastR accession: #119863) was digested with NheI and PacI. N-terminal 3CL^pro^ insert fragments with vector overhangs were generated with hygro-P-for and 3CL^pro^-*mut-x*-rev. C-terminal 3CL^pro^ insert fragments with vector overhangs were generated with 3CL^pro^-*mut-x*-for and P-hygro-rev. Double mutants were cloned by repeating the site directed mutagenesis with a second primer pair in combination with Gibson assembly on an already mutant-bearing plasmid. Cloning primers for 3CL^pro^-Off and -On mutant variants are shown in **table S13**.

### Cell lines

BHK-21 cells (American Type Culture Collection, ATCC) were cultured in Glasgow Minimum Essential Medium (GMEM) (Lonza) supplemented with 10% fetal calf serum (FCS), 5% tryptose phosphate broth, and 100 units/ml penicillin and 0.1 mg/ml streptomycin (P/S) (Gibco). 293 T cells (293tsA1609neo, ATCC), and 293-VSV (293 expressing N, P-GFP and L of VSV) (*52*) were cultured in Dulbecco’s Modified Eagle Medium (DMEM) supplemented with 10% FCS, P/S, 2% glutamine, 1x sodium pyruvate and 1x non-essential amino acids (Gibco). Vero E6 (ATCC CRL-1586) were cultured in DMEM supplemented with 5% FCS (VWR) and 1% penicillin−streptomycin−glutamine (PSG) solution (Corning). A549-hACE2 (Biomedical Resource Ontology NR-53821) were grown in DMEM supplemented with 4 mM L-glutamine, 4500 mg/l glucose, 1 mM sodium pyruvate, 1500 mg/l sodium bicarbonate, 10% FCS, 1x non-essential amino acid solution (Gibco) and 100 μg/ml blasticidin (Gibco).

### Virus recovery

VSV-G-3CL^pro^-L was rescued in 293 T cells by CaPO_4_ transfection of whole-genome VSV plasmids together with T7-polymerase, N-, P-, M-, G- and L expression plasmids as helper plasmids (*53*). Briefly, genome and helper plasmids were transfected into 293 T in the presence of 10 μM chloroquine to avoid lysosomal DNA degradation. After 6 to 16 hours, chloroquine was removed and cells were cultured until cytopathic effects occurred. M and G proteins were used as helper plasmids; although these proteins are optional in the recovery of VSV, they were chosen here as a precaution to support the rescue of a potentially attenuated virus variant. After the rescue, viruses were passaged on 293-VSV cells and plaque purified twice on BHK-21 cells. ∆P and ∆L VSV variants expressing dsRed were produced on replication supporting 293-VSV cells. VSV-G-3CL^pro^-L was fully replication competent and produced on BHK-21 cells.

### Replication kinetics, TCID_50_ assays, and dose responses

Initial replication kinetics (wild-type versus F305L) were performed as single-step kinetics. 10^5^ BHK-21 cells per well were seeded in 24-well plates one day before infection. Cells were infected in duplicate with an MOI of 5 of VSV 3CL^pro^ wild-type or the F305L variant. One hour after infection, the medium was removed, cells were washed with phosphate-buffered saline (PBS), and fresh medium was added. Supernatant was collected at the indicated time points and stored at −80°C until further analysis. For quantification, TCID_50_ assays were performed as described previously (*54*). In short, 100 μl of serial dilutions of virus were added in octuplicates to 10^3^ BHK-21 cells seeded in a 96-well plate. Six days after infection, the TCID_50_ values were read out and titers were calculated according to the Kaerber method (*55*).

For wild-type versus different mutants replication kinetics, multi-step growth kinetics were performed. 10^5^ BHK-21 cells per well were seeded in 24-well plates one day before infection. Cells were infected in duplicates with an MOI of 0.5 of VSV 3CL^pro^ wild-type or mutant variants.

For initial dose response experiments, 5 x 10^4^ BHK-21 cells per well were seeded in 48-well plates one day before infection. Cells were infected in duplicates with a MOI of 0.05 of VSV 3CL^pro^ wild-type or mutant variants and indicated concentrations of nirmatrelvir were added to the wells. After 48 hours, supernatants were collected and titrated to determine the TCID_50_.

For mutant comparing dose response experiments, 5 x 10^4^ BHK-21 cells per well were seeded in 48-well plates one day before infection. Cells were infected in duplicates with an MOI of 0.05 of VSV 3CL^pro^ wild-type or mutant variants and indicated concentrations of nirmatrelvir added to the wells. To prevent initial escape or further mutation in wild-type or already mutation-bearing viruses (“intra-assay mutants”), respectively, supernatants of all viruses were collected after the first mutant (Q192R, F305L) showed a massive cytopathic effect at 100 μM nirmatrelvir (at about 24 hours after infection). Initial dose responses (wild-type versus F305L) were performed as described above, but the supernatant was collected after 48 hours.

### Viral RNA isolation and 3CL^pro^ sequencing

VSV-G-3CL^pro^-L RNA was isolated with E.Z.N.A. Viral RNA Kit (Omega Bio-Tek Inc.) or NucleoSpin RNA Virus (Macherey-Nagel GmbH). BHK-21 cells were infected with VSV-G-3CL^pro^-L wild-type and F305L (3CLpro) mutant in 96-well plates. Virus-containing supernatants were collected from individual 96-wells and the RNA was purified from the supernatants according to manufacturers’ instructions. Then, cDNA was synthesized from isolated viral RNA by RevertAid RT Reverse Transcription Kit (Thermo Fisher Scientific). 3CL^pro^ sequence was amplified by PCR with primers (for: CTCAGGTGTTCGAACATCCTCAC and rev: GATGTTGGGATGGGATTGGC) and sent for sequencing (MicroSynth AG). Obtained sequences were mapped to the 3CL^pro^-wt (Wuhan-1) reference sequence in Geneious Prime 2022.0.2 and examined for mutations.

### Mutation selection assay

10^4^ BHK-21 cells per well in a 96-well plate were seeded one day before infection with wild-type VSV-G-3CL^pro^-L or VSV-G-3CL^pro^-L-F305L at an MOI of 0.01 and indicated nirmatrelvir doses. Each virus variant occupied 48 wells of the 96-well plate. Wells that displayed cytopathic effect after two days (25 out of 48 from parental wild-type and 17 out of 48 from parental F305L) were further passaged with increasing concentrations of nirmatrelvir with each passage (wild-type: 30, 40 and 50 μM; F305L: 50, 75 and 100 μM). **Table S1** indicates at which passage a pure mutant virus could be distinguished by Sanger sequencing, such that only one base-pair peak appeared in the chromatogram instead of a mixture with the parental virus. Only pure mutants are displayed in **Fig. 2** and **table S1**.

### Expression and purification of his-tagged 3CL^pro^ and point mutations

Plasmids containing cDNA of SARS-CoV-2 main protease 3CL^pro^ (pMCSG92 (*56*)) and mutants thereof were prepared as described in the following. Plasmids were cloned by site-directed mutagenesis with primers of **table S13** on pMCSG92. 100 ng of each plasmid was applied to 50 μl of thawed competent BL21(DE3) TUNER *E. coli* (Merck) on ice in 1.5 ml tubes. Bacterial suspensions containing the plasmids were flicked and incubated for 30 minutes on ice. Subsequently, bacteria were heated to 42°C for 90 seconds in a thermomixer without shaking, and put back on ice for 5 minutes. 400 μl of NZCYM Medium produced in-house (NZ amine (Art.-Nr. CP76.1, Roth) 10.0 g, NaCl 5.0 g, casamino acids (Gibco) 1.0 g, yeast extract (Art. Nr. 2363.2, Roth) 5.0 g, MgSO_4_ x 7 H_2_O 2.0 g, ddH_2_O to 1 L, adjusted to pH 7.4) was added to each bacterial suspension, and the bacteria were amplified for 1 hour at 37°C in a bacterial shaker in 1.5 ml tubes. Meanwhile, LB agar plates containing selection antibiotics (ampicillin) were prepared and incubated at room temperature. 200 μl of bacterial culture was crossed out on individual plates and incubated overnight at 37°C. Single colony formation was observed the following day.

Individual colonies were picked and placed in 5 ml of NZCYM medium supplemented with selection antibiotics, and amplified overnight at 37°C. The next day, overnight cultures were amplified in 1 L of NZCYM medium supplemented with selection antibiotics to an optical density (OD) of 0.2, after which protein expression was induced by applying 1 mM isopropyl-β-D-thiogalactopyranosid (IPTG). After 5 hours bacteria were harvested by centrifugation and the supernatant discarded. Bacterial pellets were frozen at −20°C for further use.

Pellets were suspended in 10 ml of Ni-NTA running buffer (20 mM Tris-HCl, 300 mM NaCl, 10 mM Imidazol, adjusted to pH 7.4) and transferred into 50 ml tubes. Bacteria were lysed using an ultrasonic probe on ice. The homogenates were centrifuged at 10,000 x g for 10 minutes and the supernatant was filtered using 0.45 μm and 0.22 μm syringe filter units. After preparing a Ni-NTA agarose column (Invitrogen, Ni-NTA Agarose R90115) and washing with 30 ml Ni-NTA running buffer, the filtered homogenate was applied to the column, and the flow-through was collected. The column was again washed with 3 x 10 ml of Ni-NTA running buffer. The His-tagged protein bound to the Ni-NTA resin, and was then eluted with 3 x 1 ml of Ni-NTA elution buffer (20 mM Tris-HCl, 300 mM NaCl, 200 mM imidazol, adjusted to pH 7.4). The obtained protein solutions were dialyzed at 4°C overnight against a storage buffer (Tris-HCl 1 mM, NaCl 4 mM, KCl 2.2 mM, TWEEN-20 0.04 vol-%, DTT 3 mM, glycerol 20.2 vol-%, adjusted to pH 8).

Eluted protein samples were further purified using size exclusion chromatography (SEC) with fast protein liquid chromatography (FPLC) (ÄKTA Pure FPLC System, Superdex 200 10/300 GL). At each step of the protocol, samples for SDS-PAGE analysis were obtained, and the successful expression of the 3CL^pro^ proteins was monitored by SDS-PAGE and Coomassie R staining. The final degree of protein purity was estimated to be greater than 90% based on Coomassie R staining, similar among the different preparations of 3CL^pro^ wild-type and mutant forms.

### Screening assay with Fluorospot read-out

3 x 10^5^ cells were seeded per well in 6-well plates and transfected one day after seeding with 3CL^pro^ plasmids using TransIT-^PRO^ (Mirus Bio LLC) and incubated overnight. Then, cells were seeded into a 96-well plate with 2 x 10^4^ cells per well in 50 μl complete growth medium. Directly after seeding, compounds and virus (MOI 0.1) were added in 50 μl complete growth medium to wells. After 48 to 72 hours, supernatants were removed, and fluorescent spots counted in a Fluoro/ImmunoSpot counter (CTL Europe GmbH). Longer incubation times of 72 hours increased the overall signal and were chosen in order to achieve a clear signal of the more resistant double mutants, which as expected have a lower signal output in 3CL^pro^-On assays. The manufacturer-provided software CTL switchboard 2.7.2. was used to scan 90% of each well area concentrically to exclude reflection from the well edges, and counts were normalized to the full area. Automatic fiber exclusion was applied while scanning. The excitation wave length for dsRed was 570 nm, the D_F_R triple band filter was used to collect fluorescence. In addition, manual quality control for residual fibers was performed. To increase comparability between 3CL^pro^-On and -Off signals, we normalized dsRed events with the following strategies. In 3CL^pro^-On, the highest compound concentrations would not reach the same value due to the different response of each mutant. Therefore, we normalized to the highest mean of the experiment, which was the wild-type signal. In 3CL^pro^-Off, untreated wells reached the same signal yield in wild-type and mutants. Therefore, we normalized the signal to each individual highest mean of the construct.

### Screening assay with flow cytometry read-out

3 x 10^5^cells were seeded per well in 6-well plates and transfected with 3CL^pro^ plasmids using TransIT-^PRO^ (Mirus Bio LLC) and incubated overnight. Then, cells were seeded into a 96-well plate with twenty thousand cells per well in 50 μl. Compound and virus (MOI 0.1) were added in 50 μl to reach desired concentrations. After two days, cells were detached with 0.05% Trypsin-EDTA (Gibco) and transferred to a 96-well round-bottom plate (TPP Techno Plastic Products AG) for automatic sampling by flow cytometry using a BD FACSCanto II. Gates were set to distinguish live and dead cells and to exclude doublets. Singlet cells were divided into dsRed positive and negative based on reference to samples, which were infected, but not treated with inhibitor (*17*). Samples were analyzed using BD FACSDiva 8.0.1 (BD Biosciences).

### Cross validation with orthologous cellular Src-3CL^pro^-tat-Luc assay

3x10^6^ 293 T were seeded per well in a 6-well dish. 24 hours later, they were transfected with 2 μg of the wild-type Src-3CL^pro^-Tat-Luc or mutants thereof with TransIT-LT1 (Mirus, catalog number MIR 2304). Four hours after transfection, cells were washed with PBS, trypsinized, resuspended in medium and counted. 2x10^5^ cell per well were seeded in 50 μl medium in a flat-bottom 96-well plate (Greiner). Inhibitor dilution series were added in 2-fold excess to required concentrations in 50 μl medium. After 44 hours, medium was removed and 50 μl of Bright-Glo reagent (Promega) added to each well. Cells were incubated for five minutes in the dark and then transferred to a white flat 96-well plate (CLS3600, Corning) for measuring luminescence on a Synergy H1 plate reader (Agilent). The percent inhibition was calculated with the following formula.

%inhibition=100−(100/relative luminescence)

### 3CL^pro^ enzymatic activity

Wild type and variant proteases were produced in-house as described in 3CL^pro^ purification method section. Solution of wild type 3CL^pro^ and variants at 85 ng / 30 μl were prepared in appropriate buffer (20 mM Tris/HCl pH 8, 150 mM NaCl, 0.1 mg/ml BSA, 1 mM DTT) and these 30 μL were added to each well in a black 96-well plate (BPS Biosciences) to get a final concentration of 50 nM in 50 μl/well. The substrate Ac-Abu-Tle-Leu-Gln↓MCA (Acetyl-L-α-aminobutyroyl-L-tert-leucyl-L-leucyl-L-glutamine α-(4-methylcoumaryl-7-amide)) was purchased from Peptide Inc., resuspended in dimethyl sulfoxide at 5 mM concentration. 20 μl buffer with diluted substrate was then added to the protein solutions at different concentrations. The plate was immediately placed inside in a GloMax Explorer reader (Promega) and fluorescence emission measured with a substrate-appropriate filter.

To determine enzymes initial velocities, we plotted RFU (relative fluorescent units) on the y-axis and time (min) on the x-axis. We performed a simple linear regression analysis. Fitting values from zero up to 60 minutes were used in a range that had a linear increase. The resulting slopes represented the initial velocity expressed as RFU/min for each protein variant at each substrate concentration. Slope values were plotted (y-axis) against the substrate concentration (x-axis). Finally, the obtained values were fitted using the “Michaelis-Menten” equation built-into GraphPad Prism 9 to extrapolate the kinetic parameters K_m_ and V_max_.

$$Y=\frac{V_{\max*}X}{K_{m}+X}$$

k_cat_ was calculated dividing V_max_ by [E_T_], where [E_T_] is the give enzyme concentration. Wild type and variant 3CL^pro^ catalytic efficiencies were determined as k_cat_/K_M_.

### Cross validation with biochemical 3CL^pro^ inhibition assay

The biochemical assay used to confirm mutations was based on the 3CL^pro^ activity assay from BPS Biosciences, catalogue number #78042–2. The 3CL^pro^ in the kit was replaced by an in-house produced 3CL^pro^ and mutants thereof, as described in 3CL^pro^ purification. Solutions of wild-type 3CL^pro^ and mutants at 5 ng/μl in 30 μl buffer (composition described above) were prepared according to the kit’s manual. Ten μl of five-fold excess to tested nirmatrelvir concentrations were added to the 30 μl of 3CL^pro^ solution and incubated for 30 minutes. Then, 10 μl of fluorogenic substrate (DABCYL-KTSAVLQSGFRKME-EDANS) was added (generating a in total a 1:5 dilution of the excess nirmatrelvir and therefore final concentrations) and incubated for 4 hours. Fluorescence was induced with 365 nm UV-light and read at 460 nm in a Glomax Explorer (Promega). Blanks (assay buffer plus substrate) values were subtracted from sample values.

### Replication kinetics with recombinant SARS-CoV-2 expressing mCherry

Monolayers of Vero E6 cells (6-well plate, 10^6^ cells per well, triplicates) were infected with the indicated viruses at MOI 0.01. After viral absorption for 3 hours at 37°C, the supernatant was discarded, the cells were washed three times with PBS, and post-infection media (3 ml/well) was added. At the indicated time points, the supernatant (300 μl/well) was collected and titrated by plaque assay (*29*).

### Cross-validation with recombinant SARS-CoV-2 (rWA1) expressing mCherry

A monolayer of A549-hACE2 cells was infected with 300 plaque-forming units (PFU) of indicated viruses in quadruplicates at 37°C. After viral adsorption for 1 hour, the supernatant was discarded and the cells were washed twice with PBS. Then, phenol red-free post-infection medium (DMEM +2% fetal bovine serum +1% penicillin-streptomycin-glutamine (PSG)) containing the indicated concentrations of nirmatrelvir was added to each well. The mCherry intensity was determined at 48 and 72 hours post-infection under a Synergy LX Multimode Reader (Agilent). Wells without drug or virus were used as negative controls or baseline signal. Positive controls were wells with virus, but no drug. Infection percentages of wells with different amounts of inhibitor were calculated by subtracting the negative control (mean of wells without virus or drug) and then dividing by the positive control (mean of wells with virus but without drug). Data were analyzed in GraphPad Prism 9 and IC_50_ values were calculated as the highest dilution of the nirmatrelvir-containing sample that prevents 50% plaque formation in infected cells, determined by a sigmoidal dose-response curve (see statistical analysis section).

### IC_50_ and EC_50_ calculations

In this study, different assay systems were used to generate resistance data, namely VSV-based cellular assays with FluoroSpot and flow cytometry read-outs, an orthologous cell-based assay with a luciferase read-out, as well as a biochemical assay and SARS-CoV-2-mCherry assay with fluorescence read-outs. Although the magnitudes of resistance are different in these assays, the tendencies agree. We expected the dynamic range of the 3CL^pro^ cellular assays to be greater than in a biochemical assay, where there is a fixed amount of enzyme. In cells, the continuous renewal of protease-viral fusion proteins in an excess of inhibitor likely led to a later plateauing of the signal. At lower concentrations, compound molecules are depleted and the signal plateaus. In FluoroSpot read-outs, the 3CL^pro^-On assay data were normalized to the highest mean value in an experiment. 3CL^pro^-Off data were normalized to the highest value of each construct in an experiment. In the flow cytometry experiments, 3CL^pro^-On assay data were also normalized to the highest value of each construct in an experiment. For purified wild-type and mutant enzymes, IC_50_ values were determined using the biochemical assay “3CL Protease, Untagged (SARS-CoV-2)” from BPS Biosciences with the assays substrate DABCYL-KTSAVLQSGFRKME-EDANS. IC_50_ and EC_50_ calculations and statistical analysis for all assays were performed with GraphPad Prism 9 (see statistical analysis section).

### Nanopore sequencing of recombinant SARS-CoV-2 (rWA1) expressing mCherry

To validate the sequence of the recombinant SARS-CoV-2 (rWA1) expressing mCherry, we used the Nanopore sequencing “Midnight protocol”, version 6 (*57*). Primer pools generating 1200 bp overlapping amplicons were purchased from Integrated DNA Technologies, as referenced in the abovementioned protocol. The sequencing reactions were prepared using the Rapid Barcoding Kit SQK-RBK110.96 (Oxford Nanopore Technologies) and were performed in a sequencer (MinION Mk1B) using a proprietary flowcell (R9.4.1, Oxford Nanopore Technologies). Electrical signals are translated into nucleotide sequences (basecalling). Sequenced reads were sorted into separate files for each sample (demultiplexing). Demultiplexing was done using the super high accuracy model in Guppy 6.1.5. Output sequences generated so called fastq files and sequences below 200 and above 1200 bp were removed. Sequences between 200 and 1200 bp were assembled with the algorithm epi2me-labs/wf-artic v0.3.18 in Nextflow 22.04.4. The SARS-CoV-2 lineage pangolin 4.1.1 was used to map the sequences. A visualization application (Nextclade 2.4.0) was used to check mutations.

### Protein structure preparation for molecular modelling

The three-dimensional structure of the SARS-CoV-2 3CL^pro^ complexed with nirmatrelvir was retrieved from the Protein Data Bank (PDB code: 7RFW, method: X-ray diffraction, resolution: 1.73 Å (*31*)) and prepared for molecular modeling evaluations exploiting several tools implemented in the Molecular Operating Environment (MOE) 2022.02 suite (*16*). Specifically, the “Structure Preparation” tool was used to assign each protein residue with alternative conformations to the one characterized by the highest occupancy value, and the “Protonate 3D” program was exploited to assign each titratable amino-acid to the most appropriate protonation state at a pH of 7.4. Finally, the coordinates of hydrogen atoms were energy minimized using the AMBER10:EHT force field (*58*) until a gradient of 0.1 kcal mol^−1^ Å^−2^ was reached.

### In silico alanine and resistance mutation scanning

An in silico evaluation of the impact of SARS-CoV-2 3CL^pro^ mutations on both the stability of the protein and the affinity towards nirmatrelvir was conducted using the “Protein Design” module of MOE, using the previously mentioned complex structure. Particularly, the “Alanine Scan” and “Resistance Scan” tools were used to perform two virtual mutagenesis experiments.

First, we applied the “Alanine Scan” interface, in which each of the 612 amino acids composing the dimeric SARS-CoV-2 3CL^pro^ was mutated into an alanine residue, calculating at each given time the energy difference between the mutated protein and the wild-type form concerning both the potential energy of the protein itself (dStability) and the affinity towards nirmatrelvir (dAffinity). Values were then color plotted on the crystal complex of nirmatrelvir and SARS-CoV-2 3CL^pro^ using UCSF Chimera (*59*). Then we used the “Resistance Scan” interface to investigate the impact of a selected pool of mutations: Y54C, G138S, L141F, L167F, and Q192R. For both types of calculations, the conformational sampling was carried out through LowModeMD (*60*), using the AMBER10:EHT forced field coupled with the Generalized Born implicit solvent model (*61*); the dAffinity value was determined through the GBVI/VSA (*62*) method.

### Statistical analysis

Raw and normalized data are provided in **Data file S4**. Dose response data points of 3CL^pro^-On, Off and biochemical assays were fitted using a four-parameter logistic regression (sigmoid, 4PL, X is concentration). IC_50_ values were extrapolated as the concentration value at which the signal was 50% between the top and bottom plateaus of each sub-dataset. Dose responses curves of the Src-3CL^pro^-Tat-Luc-based assay were fitted with the same regression, setting X as 2 for IC_50_ extrapolation.

$$Y=\mathrm{Bottom}+\frac{\mathrm{Top}-\mathrm{Bottom}}{1+{\left({\mathrm{IC}}_{50}/X\right)}^{\mathrm{HillSlope}}}$$

Data obtained with flow cytometry were normalized and fit-based using the non-linear regression function “[Agonist] vs. normalized response”. The EC_50_ values were extrapolated as the medium value between the top and bottom plateaus of each sub-dataset.

$$Y=\frac{100*X}{{\mathrm{EC}}_{50}+X}$$

Nirmatrelvir dose response curves of recombinant SARS-CoV-2 expressing mCherry were normalized and fitted using the non-linear regression function “log(inhibitor) vs. normalized response - variable slope”.

$$Y=\frac{100}{1+10^{\log(\mathrm{IC}50\text{-}X)*\mathrm{HillSlope}}}$$

Kinetic parameters and catalytic activity of wild-type and mutant 3CL^pro^ enzymes were calculated as described in the corresponding method section. 95% confidence intervals were generated by the described fittings and are provided in supplementary tables together with IC_50_ and EC_50_ values. All statistical analyses were performed with GraphPad Prism 9.

## Supplementary Materials

### Other Supplementary Material for this manuscript includes the following:

MDAR Reproducibility Checklist

## Notes

View/request a protocol for this paper from *Bio-protocol*.
