# Supplementary material for: SARS-CoV-2 3CLpro mutations selected in a VSV-based system confer resistance to nirmatrelvir, ensitrelvir, and GC376
Source: Sci Transl Med. 2022 Oct 4:eabq7360. doi: 10.1126/scitranslmed.abq7360 (PMC9765458; doi:10.1126/scitranslmed.abq7360)
Supplement: Supplementary file 1 — Fig. S1 to S11 Table S1 to S13 [file scitranslmed.abq7360_sm.pdf]

Science Translational Medicine

Supplementary Materials for

SARS-CoV-2 3CL^{pro} mutations selected in a VSV-based system confer resistance to nirmatrelvir, ensitrelvir, and GC376

Emmanuel Heilmann *et al.*

Corresponding author: Emmanuel Heilmann, Emmanuel.Heilmann@i-med.ac.at

Sci. Transl. Med. **0**, eabq7360 (2022)
DOI: 10.1126/scitranslmed.abq7360

The PDF file includes:

Figs. S1 to S11
Tables S1 to S13
Legends for data files S1 to S4

Other Supplementary Material for this manuscript includes the following:

Data files S1 to S4

Supplemental Figures and Tables

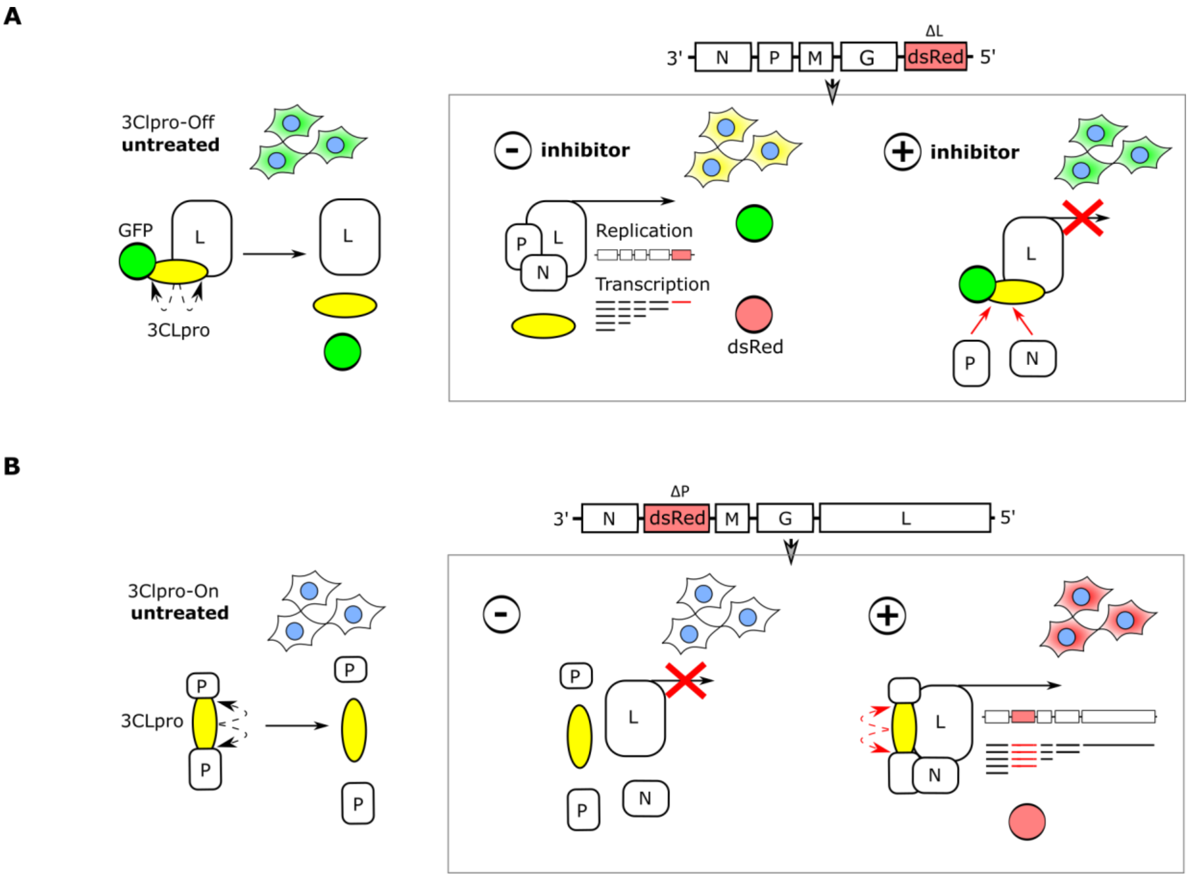

Fig. S1. Design principles of the 3CL^{pro}-Off and -On systems for quantifying 3CL^{pro} activity and chemical inhibition. (A) Schematics of the assay comprising VSV-ΔL-dsRed and GFP-3CL^{pro}-L. Transfection of GFP-3CL^{pro}-L plasmid yields GFP-positive cells, and subsequent infection with VSV-ΔL-dsRed particles yields GFP/dsRed-positive cells (yellow in merge). Addition of a 3CL^{pro} inhibitor suppresses the accumulation of dsRed signal (3CL^{pro}-Off system). **(B)** Schematics of the assay comprising VSV-ΔP-dsRed and P:3CL^{pro}. Co-expression of these two vectors in the same cells yields no virus replication due to VSV P protein auto-cleavage by 3CL^{pro}. Treatment of these cells with a 3CL^{pro} inhibitor restores P protein function and enables virus replication (3CL^{pro}-On system).

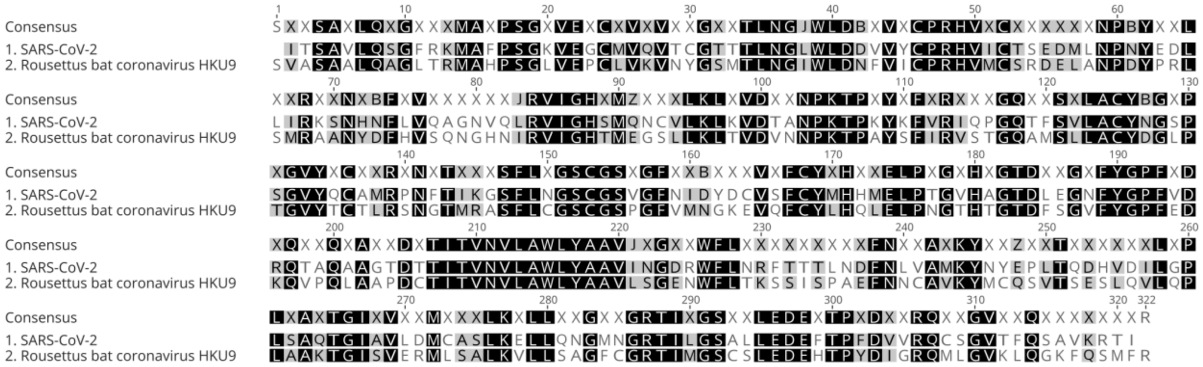

Fig. S2. Sequence identity of SARS-CoV-2 and MHV 3CL^{pro} amino acid sequence. MUltiple Sequence Comparison by Log-Expectation (MUSCLE) sequence alignment of SARS-CoV-2 and mouse hepatitis virus (MHV) 3CL^{pro} shows 50% identity of amino acid sequences.

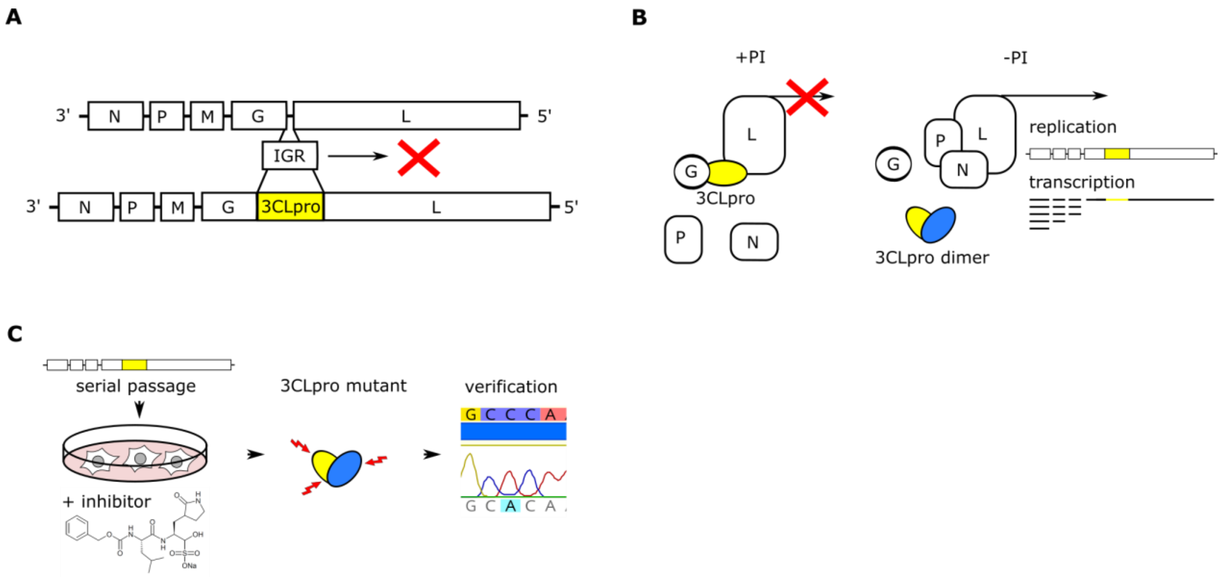

Fig. S3. Design principles of the VSV-3CL^{pro} system for selecting 3CL^{pro} mutants. (A) The genomic scheme of VSV is shown; the intergenic region (IGR) between the glycoprotein (G) and the polymerase (L) was replaced with the SARS-CoV-2 3CL^{pro}. **(B)** Addition of a protease inhibitor (PI) stalls VSV G, 3CL^{pro}, and L in a non-functional polyprotein. In the absence of the PI, VSV can replicate and transcribe genes**. (C)** Serial passage of VSV-G-3CL^{pro}-L in the presence of a 3CL^{pro} inhibitor can lead to resistance mutations.

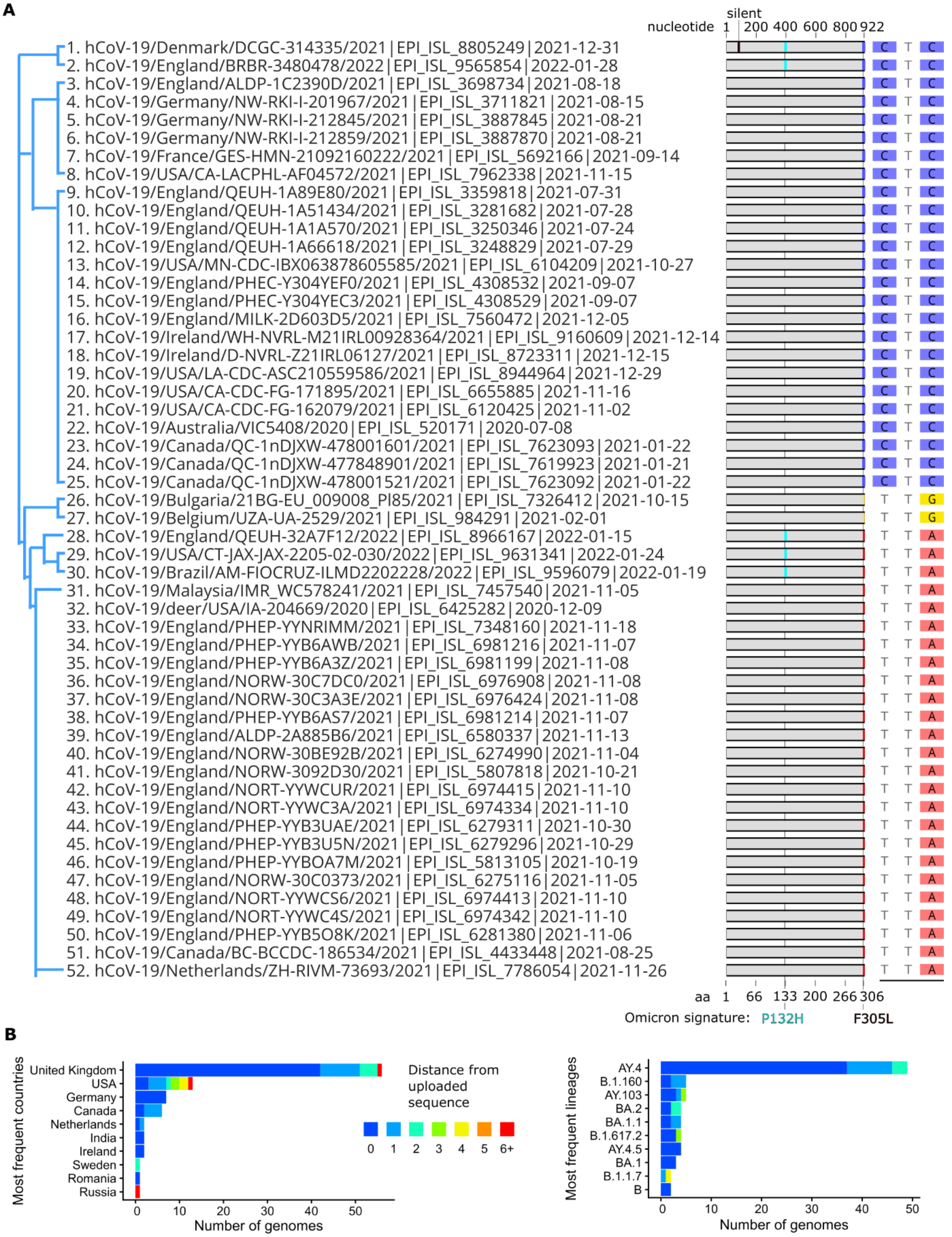
A
silent
nucleotide 1 200 400 600 800 922
1. hCoV-19/Denmark/DCGC-314335/2021|EPI_ISL_8805249|2021-12-31
2. hCoV-19/England/BRBR-3480478/2022|EPI_ISL_9565854|2022-01-28
3. hCoV-19/England/ALDP-1C2390D/2021|EPI_ISL_3698734|2021-08-18
4. hCoV-19/Germany/NW-RKI-I-201967/2021|EPI_ISL_3711821|2021-08-15
5. hCoV-19/Germany/NW-RKI-I-212845/2021|EPI_ISL_3887845|2021-08-21
6. hCoV-19/Germany/NW-RKI-I-212859/2021|EPI_ISL_3887870|2021-08-21
7. hCoV-19/France/GES-HMN-21092160222/2021|EPI_ISL_5692166|2021-09-14
8. hCoV-19/USA/CA-LACPHL-AF04572/2021|EPI_ISL_7962338|2021-11-15
9. hCoV-19/England/QEUH-1A89E80/2021|EPI_ISL_3359818|2021-07-31
10. hCoV-19/England/QEUH-1A51434/2021|EPI_ISL_3281682|2021-07-28
11. hCoV-19/England/QEUH-1A1A570/2021|EPI_ISL_3250346|2021-07-24
12. hCoV-19/England/QEUH-1A66618/2021|EPI_ISL_3248829|2021-07-29
13. hCoV-19/USA/MN-CDC-IBX063878605585/2021|EPI_ISL_6104209|2021-10-27
14. hCoV-19/England/PHEC-Y304YEF0/2021|EPI_ISL_4308532|2021-09-07
15. hCoV-19/England/PHEC-Y304YEC3/2021|EPI_ISL_4308529|2021-09-07
16. hCoV-19/England/MILK-2D603D5/2021|EPI_ISL_7560472|2021-12-05
17. hCoV-19/Ireland/WH-NVRL-M21IRL00928364/2021|EPI_ISL_9160609|2021-12-14
18. hCoV-19/Ireland/D-NVRL-Z21IRL06127/2021|EPI_ISL_8723311|2021-12-15
19. hCoV-19/USA/LA-CDC-ASC210559586/2021|EPI_ISL_8944964|2021-12-29
20. hCoV-19/USA/CA-CDC-FG-171895/2021|EPI_ISL_6655885|2021-11-16
21. hCoV-19/USA/CA-CDC-FG-162079/2021|EPI_ISL_6120425|2021-11-02
22. hCoV-19/Australia/VIC5408/2020|EPI_ISL_520171|2020-07-08
23. hCoV-19/Canada/QC-1nDJXW-478001601/2021|EPI_ISL_7623093|2021-01-22
24. hCoV-19/Canada/QC-1nDJXW-477848901/2021|EPI_ISL_7619923|2021-01-21
25. hCoV-19/Canada/QC-1nDJXW-478001521/2021|EPI_ISL_7623092|2021-01-22
26. hCoV-19/Bulgaria/21BG-EU_009008_PI85/2021|EPI_ISL_7326412|2021-10-15
27. hCoV-19/Belgium/UZA-UA-2529/2021|EPI_ISL_984291|2021-02-01
28. hCoV-19/England/QEUH-32A7F12/2022|EPI_ISL_8966167|2022-01-15
29. hCoV-19/USA/CT-JAX-JAX-2205-02-030/2022|EPI_ISL_9631341|2022-01-24
30. hCoV-19/Brazil/AM-FIOCRUZ-ILMD2202228/2022|EPI_ISL_9596079|2022-01-19
31. hCoV-19/Malaysia/IMR_WC578241/2021|EPI_ISL_7457540|2021-11-05
32. hCoV-19/deer/USA/IA-204669/2020|EPI_ISL_6425282|2020-12-09
33. hCoV-19/England/PHEP-YYNRIMM/2021|EPI_ISL_7348160|2021-11-18
34. hCoV-19/England/PHEP-YYB6AWB/2021|EPI_ISL_6981216|2021-11-07
35. hCoV-19/England/PHEP-YYB6A3Z/2021|EPI_ISL_6981199|2021-11-08
36. hCoV-19/England/NORW-30C7DC0/2021|EPI_ISL_6976908|2021-11-08
37. hCoV-19/England/NORW-30C3A3E/2021|EPI_ISL_6976424|2021-11-08
38. hCoV-19/England/PHEP-YYB6AS7/2021|EPI_ISL_6981214|2021-11-07
39. hCoV-19/England/ALDP-2A885B6/2021|EPI_ISL_6580337|2021-11-13
40. hCoV-19/England/NORW-30BE92B/2021|EPI_ISL_6274990|2021-11-04
41. hCoV-19/England/NORW-3092D30/2021|EPI_ISL_5807818|2021-10-21
42. hCoV-19/England/NORT-YYWCUR/2021|EPI_ISL_6974415|2021-11-10
43. hCoV-19/England/NORT-YYWC3A/2021|EPI_ISL_6974334|2021-11-10
44. hCoV-19/England/PHEP-YYB3UAE/2021|EPI_ISL_6279311|2021-10-30
45. hCoV-19/England/PHEP-YYB3U5N/2021|EPI_ISL_6279296|2021-10-29
46. hCoV-19/England/PHEP-YYBOA7M/2021|EPI_ISL_5813105|2021-10-19
47. hCoV-19/England/NORW-30C0373/2021|EPI_ISL_6275116|2021-11-05
48. hCoV-19/England/NORT-YYWCS6/2021|EPI_ISL_6974413|2021-11-10
49. hCoV-19/England/NORT-YYWC4S/2021|EPI_ISL_6974342|2021-11-10
50. hCoV-19/England/PHEP-YYB5O8K/2021|EPI_ISL_6281380|2021-11-06
51. hCoV-19/Canada/BC-BCCDC-186534/2021|EPI_ISL_4433448|2021-08-25
52. hCoV-19/Netherlands/ZH-RIVM-73693/2021|EPI_ISL_7786054|2021-11-26
aa 1 66 133 200 266 306
Omicron signature: P132H F305L
B
Most frequent countries
United Kingdom
USA
Germany
Canada
Netherlands
India
Ireland
Sweden
Romania
Russia
0 10 20 30 40 50
Number of genomes
Distance from uploaded sequence
0 1 2 3 4 5 6+
Most frequent lineages
AY.4
B.1.160
AY.103
BA.2
BA.1.1
B.1.617.2
AY.4.5
BA.1
B.1.1.7
B
0 10 20 30 40 50
Number of genomes

Fig. S4. Regional outbreaks of SARS-CoV-2-F305L. **(A)** An Unweighted Pair Group Method with Arithmetic Mean (UPGMA) tree shows 52 F305L mutant sequences from the global initiative on sharing avian flu data (GISAID) EpiCoV project. Only high-quality sequences (no deletions, insertions, stop codons, or frame shifts) were used for alignment. Turquoise lines indicate Omicron signature mutation, P132H. The column of three nucleotides to the right depict different codons for leucine. **(B)** GISAID data analysis of all F305L mutants found on July 7th, 2022.

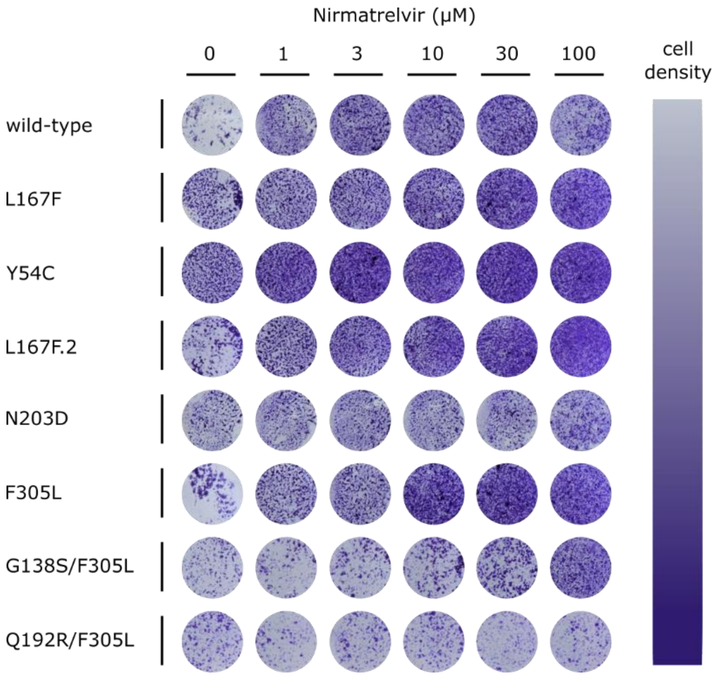

Fig. S5. Crystal violet staining of dose response experiments. Cells were stained with crystal violet after removal of supernatant for titration. Lower cell density indicates cytopathic effect.

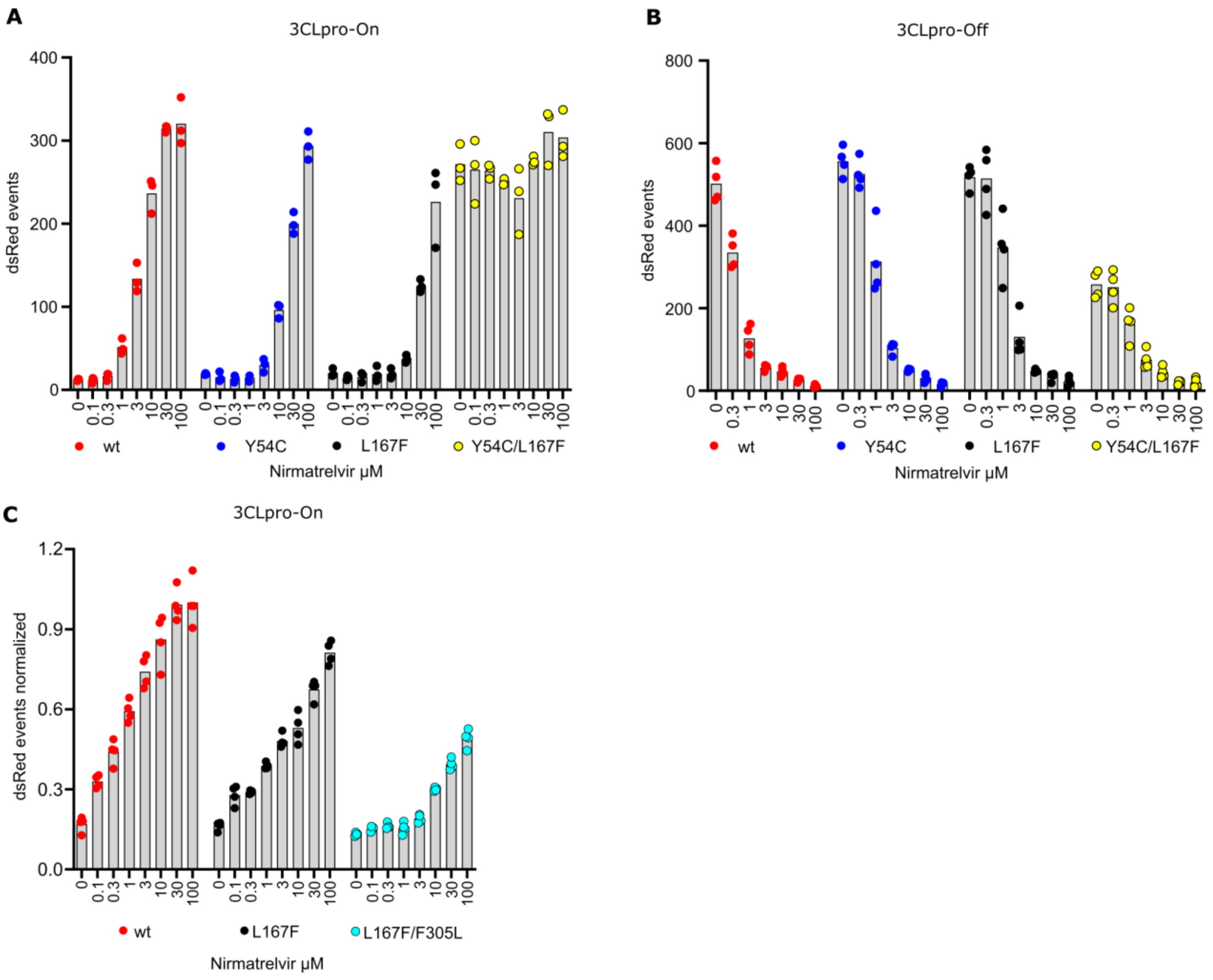

Fig. S6. Supporting data for re-introduced mutations into 3CL^{pro}-On and -Off. (A) dsRed expression in 3CL^{pro}-On wild-type versus Y54C, L167F and Y54C/L167F mutants is shown at increasing concentrations of nirmatrelvir (n = 3 biologically independent replicates per condition with average values represented by histogram bars). **(B)** dsRed expression in 3CL^{pro}-Off wild-type versus Y54C, L167F and Y54C/L167F mutants is shown at increasing concentrations of nirmatrelvir. Values were not normalized (n = 4 biologically independent replicates per condition with average values represented by histogram bars). **(C)** The dose response experiment from **Fig. 4E** results are shown as measured by flow cytometry. Values were not normalized (n = 4 biologically independent replicates per condition with average values represented by histogram bars).

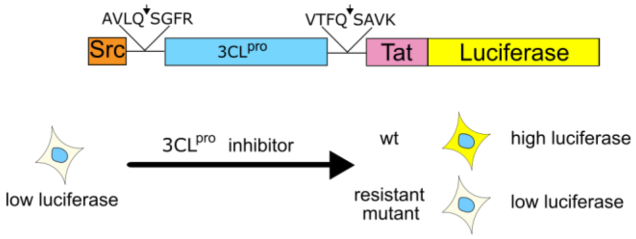

Fig. S7. Cross validation screening with orthologous cellular assay. Scheme of cellular gain-of-signal assay based on Src-3CL^{pro}-Tat-Luc polyprotein. Adding a 3CL^{pro} inhibitor leads to high fluorescence in the wt construct and lower fluorescence in mutant constructs.

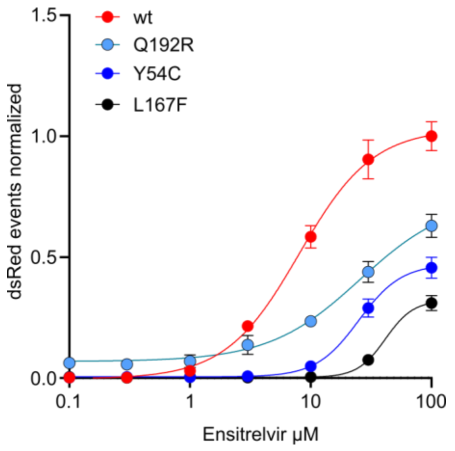

Fig. S8. Effect of Y54C, L167F and Q192R mutants on inhibition with ensitrelvir. dsRed expression was measured in 3CL^{pro}-On wild-type versus Y54C, L167F and Q192R after exposure to increasing concentrations of ensitrelvir. Data are presented as the standard deviation of n = 4 biologically independent replicates per condition.

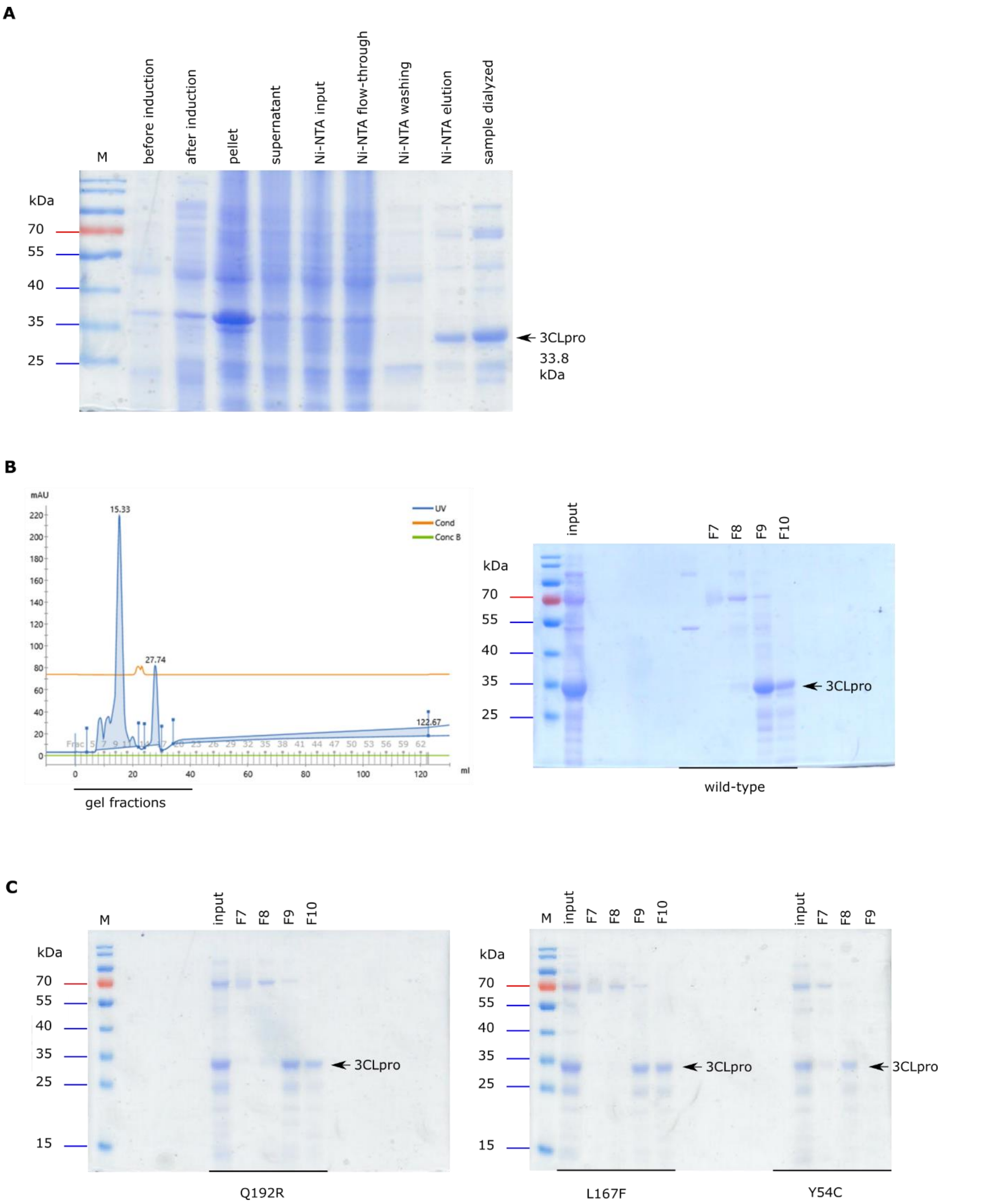

Fig. S9. 3CL^{pro} production quality control. (A) Different steps of purification are shown. Lane 1 and 2: bacterial lysates before and after induction with Isopropyl ß-D-1-thiogalactopyranoside. Lane 3: bacterial pellet. Lane 4: supernatant. Lane 5: resuspended pellet in Ni-NTA buffer. Lane 6: flow-through of Ni-NTA column. Lane 7: Ni-NTA washing flow-through. Lane 8: Ni-TNA elution. Lane 9: dialyzed sample. **(B)** A chromatogram and a polyacrylamide gel of wild-type 3CL^{pro} are shown. Fractions 9 and 10 contained 3CL^{pro} (33.8 kDa). UV: absorption at 280 nm; Cond: the conductivity of the liquid; Conc B: baseline / secondary (B) pump flow of FPLC machine. **(C)** Polyacrylamide gels are shown for mutant 3CL^{pro} enzymes.

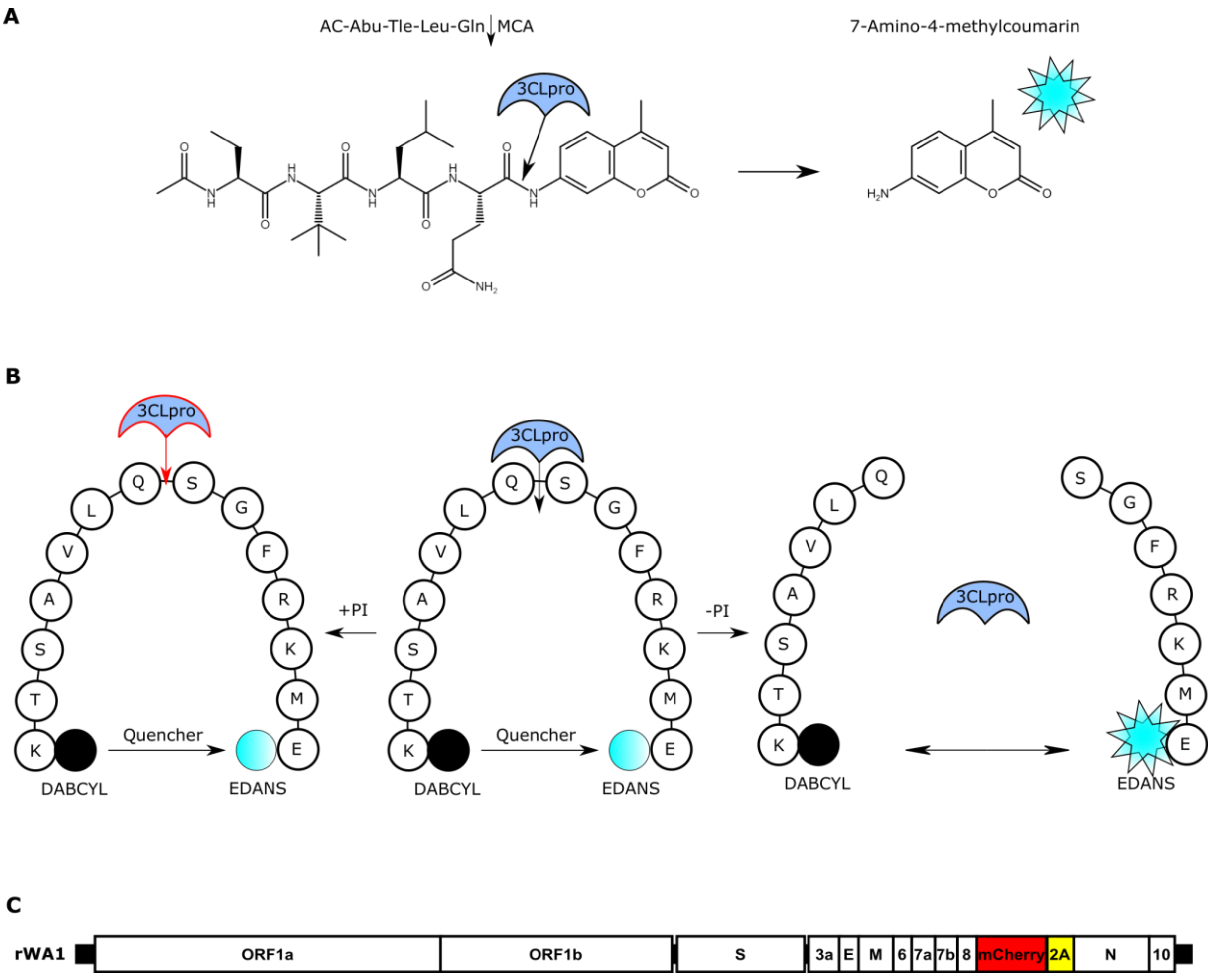

Fig. S10. Cross validation screenings with biochemical assay and SARS-CoV-2-mCherry. (A) Scheme of enzymatic assay comprising the substrate Ac-Abu-Tle-Leu-Gln↓MCA. Upon cleavage of $3CL^{pro}$, the fluorescent molecule 7-amino-4-methylcoumarin (AMC) is released. **(B)** Scheme of enzymatic assay comprising a quencher (DABCYL), the substrate peptide (KTSAVLQSGFRKME) and fluorogen (EDANS). Upon cleavage of $3CL^{pro}$, the fluorescent molecule EDANS is released. Addition of protease inhibitor (+PI) leads to sustained quenching of the fluorogen. **(C)** Genome scheme of rWA1 SARS-CoV-2-mCherry.

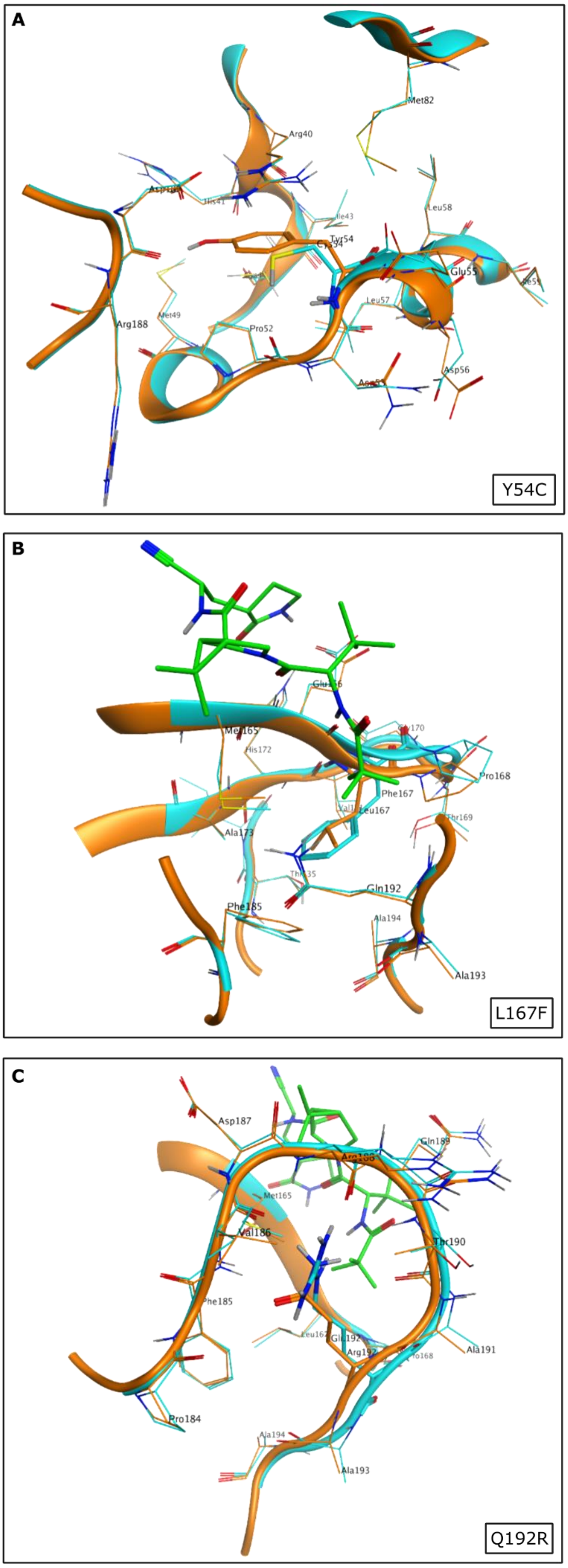

A
Met82
Arg40
Leu58
Tyr54
Glu55
Leu57
Arg188
Met49
Pro52
Asp56
Y54C
B
Met165
His172
Phe167
Leu167
Pro168
Ala173
Gln192
Phe185
Ala194
Ala193
L167F
C
Asp187
Gln189
Met165
Val186
Thr190
Phe185
Gln192
Arg192
Ala191
Pro184
Ala194
Ala193
Q192R

Fig. S11. In silico resistance mutation scanning with Molecular Operating Environment. **(A)** Superposition between the wild-type SARS-CoV-2 3CL^{pro} (PDB ID: 7RFW, orange) and the modeled structure for the Y54C mutant (cyan). **(B)** Superposition between the wild-type SARS-CoV-2 3CL^{pro} (PDB ID: 7RFW, orange) and the modeled structure for the L167F mutant (cyan). Nirmatrelvir structure is shown in green. **(C)** Superposition between the wild-type SARS-CoV-2 3CL^{pro} (PDB ID: 7RFW, orange) and the modeled structure for the Q192R mutant (cyan). Nirmatrelvir structure is shown in green

Table S1: 3CL^{pro} mutations derived from VSV-G-3CL^{pro}-L virus. GISAID coverage retrieval 2nd June 2022, excluding sequences with frame shifts. Numbers in parentheses indicate the number of times the mutants were observed.

Passage	Parental	Amino acid change	Localization	NCBI (coverage 3rd March 2022)	GISAID (coverage 2nd June 2022)
1	wt	D216Y	allosteric	D216N (16)	D216Y (9)
1	wt	G2V, D295Y	cleavage site, allosteric	G2C (13)	G2V (9), D295Y (7)
1	F305L	F3S	cleavage site	F3V (25)	F3S (3)
1	wt	L32I, H80P, D216G	allosteric, allosteric, allosteric	L32F (78), H80Y (43)	L32I (210), H80P (2), D216G (11)
1	wt	L167F	catalytic site	L167F (89)	L167F (6)
1	wt	V204F	allosteric	V204A (1013), V204I (27), V204G (15)	V204F (3)
1	wt	Y54C	catalytic site		Y54C (7)
1	F305L	Y54S	catalytic site		Y54S ()
1	wt	Q299P	dimerization interface		-
1	wt	Y118H	allosteric		Y118H (7)
2	F305L	A194S, F219S	near catalytic site, allosteric	A194S (55)	A194S (591), F219S (6)
2	F305L	D197Y	near catalytic site	D197N (14)	D197Y (31)
2	F305L	D295G	allosteric		D295G (3)
2	wt	D295N	allosteric		D295N (1)
2	F305L	F219S	allosteric		F219S (6)
2	F305L	G138S	catalytic site	G138S (14)	G138S (9)
2	wt	L208W	allosteric		L208W (2)
2,3	wt	N203D	allosteric	N203D (17)	N203D (8)
2	F305L	N203H	allosteric	N203Y (18)	N203H (10)
2	F305L	N203K	allosteric	N203K (11)	N203K (30)
2	wt	N203S	allosteric	N203T (29)	N203S (31)
2	F305L	Q192R	catalytic site		Q192R (4)
2	wt	Q299K	dimerization interface		Q299K (3)
2	F305L	R298G	dimerization interface	R298K (76), R298S (16)	R298G (273)
2	wt	C128Y, V202F	dimerization interface, allosteric		C128Y (8), V202F (32)
2	F305L	V296G	cleavage site	V296I (291)	V296G (1)
3	F305L	T98I	allosteric	T98A (10)	T98I (20)
3	wt	P99L	allosteric	P99L (17)	P99L (161)
3	wt	Q127R, Q299K	dimerization interface, dimerization interface		Q127R (4), Q299K (3)
3	wt	F305L	cleavage site	F305L (21)	F305L (101)
3	F305L	I200T, N203K	allosteric	I200N (12), N203K (11)	I200T (7), N203K (23)

3	F305L	V204F	allosteric		V204F (3)
3	wt	G2V, Y126F, D295Y	cleavage site, dimerization interface, allosteric	G2C (13)	G2V (9), Y126F (23), D295Y (7)
3	wt	D216Y	allosteric		D216Y (9)
3	wt	L141F	catalytic site		L141F (10)
3	wt	L32I, H80P, D216G, R222Q	allosteric, allosteric, allosteric, allosteric	L32F (78), H80Y (43), R222Q (17)	L32I (210), H80P (2), D216G (11), R222Q (384)

Table S2: 3CL^{pro} amino acid residues in close proximity (within 5 Å) to nirmatrelvir.

Amino acid residue	Corresponding mutants
S1	
H41	
M49	
Y54	Y54C, S
F140	
L141	L141F
N142	
G143	
S144	
C145	
H163	
H164	
M165	
E166	
L167	L167F
P168	
H172	
V186	
D187	
R188	
Q189	
T190	
A191	
Q192	Q192R

Table S3: Deposition count in GISAID data base of 3CL^{pro} mutations derived from VSV-G-3CL^{pro}-L virus before and after 22nd December 2021 (retrieval 6th June 2022, excluding sequences with frame shifts).

Amino acid change	Depositions before Paxlovid EUA (22nd December 2021)	Depositions after Paxlovid EUA
D216Y	85	17
G2V, D295Y	7, 5	5, 2
F3S	3	-
L32I, H80P, D216G	177, 3, 8	36, 4, 5
L167F	7	9
V204F	4	2
Y54C	5	5
Y54S	2	3
Q299P	-	-
Y118H	6	2
A194S, F219S	637, 8	182, 4
D197Y	18	17
D295G	3	2
D295N	3	1
F219S	8	4
G138S	12	3
L208W	2	1
N203D	21	26
N203H	3	5
N203K	11	25
N203S	31	16
Q192R	1	35
Q299K	4	3
R298G	260	16
C128Y, V202F	8, 27	3, 8
V296G	2	-
T98I	7	14
P99L	136	28
Q127R, Q299K	4, 4	6, 3
F305L	88	18
I200T, N203K	5, 11	6, 25
V204F	4	2
G2V, Y126F, D295Y	7, 46, 5	5, 33, 2
D216Y	85	17
L141F	22	12
L32I, H80P, D216G, R222Q	177, 3, 8, 198	36, 4, 5, 201

Table S4: Nirmatrelvir half-maximal inhibitor concentration (IC_{50}) values of $3CL^{pro}$-On and -Off wild-type and mutant variants.

Wild-type versus Y54C and L167F				
	$3CL^{pro}$-On IC_{50} (μM)	Fold change (versus wt)	$3CL^{pro}$-Off IC_{50} (μM)	Fold change (versus wt)
Wild-type	4.57 (3.77 - 5.60)	1	0.38 (0.30 - 0.53)	1
Y54C	22.62 (19.07 - 28.52)	5	1.00 (0.83 - 1.29)	2.6
L167F	31.92 (25.10 to 79.17)	7	1.40 (1.15 to 1.75)	3.6
Omicron (O) versus O+Y54C; O+L167F				
	$3CL^{pro}$-On IC_{50} (μM)	Fold change (versus wt)	$3CL^{pro}$-Off IC_{50} (μM)	Fold change (versus wt)
Wild-type	11.81 (9.52 - 15.63)	1	0.18 (0.16 - 0.23)	1
O+Y54C	37.75 (43.09 - 109.70)	3.2	2.95 (2.57 - 3.60)	16.5
O+L167F	58.31 (29.73 - 55.04)	5	1.49 (1.33 - 1.69)	8.3
Wild-type versus L167F; L167F+F305L				
	$3CL^{pro}$-On IC_{50} (μM)	Fold change (versus wt)	$3CL^{pro}$-Off IC_{50} (μM)	Fold change (versus wt)
Wild-type	2.68 (2.26 - 3.21)	1	0.41 (0.36 - 0.52)	1
L167F	20.08 (18.36 - 22.05)	7.5	1.43 (1.20 - 1.61)	3.5
L167F/F305L	36.36 (30.63 - 49.48)	13.5	3.77 (2.94 - 4.97)	9.2
Wild-type versus Q192R; Q192R/F305L				
	$3CL^{pro}$-On IC_{50} (μM)	Fold change (versus wt)	$3CL^{pro}$-Off IC_{50} (μM)	Fold change (versus wt)
Wild-type	4.01 (3.71 - 4.35)	1	0.79 (0.71 - 0.97)	1
Q192R	118.4 (39.20 - to high)	29.5	5.36 (4.52 - 6.78)	5.9
Q192R/F305L	141.7 (59.20 - to high)	35.3	12.5 (10.18 - 16.22)	12.5
Wild-type versus A194S; G138S				
	$3CL^{pro}$-On IC_{50} (μM)	Fold change (versus wt)	$3CL^{pro}$-Off IC_{50} (μM)	Fold change (versus wt)
Wild-type	11.38 (9.95 - 13.25)	1	0.10 (0.09 - 0.16)	1
A194S	37.82 (30.40 - 55.11)	3.3	0.49 (0.43 - 0.65)	4.9
G138S	39.3 (25.03 - 124.80)	3.5	0.27 (0.22 - 0.31)	2.7

Table S5: GC376 IC_{50} values of $3CL^{pro}$-On wild-type and mutant variants.

Wild-type versus Y54C; L167F		
	$3CL^{pro}$-On IC_{50} (μM)	Fold change (versus wt)
Wild-type	9.74 (7.98 - 12.37)	1
L167F	28.54 (26.70 - 30.98)	2.9
Y54C	34.74 (29.98 - 47.20)	3.7

Wild-type versus Q192R; Q192R/F305L		
	$3CL^{pro}$-On IC_{50} (μM)	Fold change (versus wt)
Wild-type	9.38 (8.44 - 10.46)	1
Q192R	24.43 (19.31 - 34.90)	2.6

Wild-type versus A194S; G138S		
	$3CL^{pro}$-On IC_{50} (μM)	Fold change (versus wt)
Wild-type	12.62 (11.05 - 14.69)	1
A194S	21.99 (19.50 - 25.62)	1.7
G138S	18.99 (15.12 - 26.56)	1.5

Table S6: Half maximal effective concentration (EC_{50}) values of $3CL^{pro}$-On wild-type and mutant variants as measured by flow cytometry.

Wild-type versus L167F; L167F/F305L (with FACS)		
	$3CL^{pro}$-On EC_{50} (μM)	Fold change (versus wt)
Wild-type	0.91 (0.70 - 1.19)	1
L167F	3.53 (2.39 - 5.22)	3.8
L167F/F305L	11.07 (9.35 - 13.10)	12.5

Table S7: Two-fold change (2FC) for nirmatrelvir, ensitrelvir, and GC376.

	Nirmatrelvir 2FC (nM)	Fold change (versus wt)	Ensitrelvir 2FC (nM)	Fold change (versus wt)	GC376 2FC (nM)	Fold change (versus wt)
Wild-type	11.4	1	17.1	1	145.3	1
L167F	96.2	8.5	284.3	16.6	570.2	3.9

Table S8: Ensitrelvir IC_{50} values of $3CL^{pro}$-On wild-type and mutant variants.

	Ensitrelvir IC_{50} (µM)	Fold change (versus wt)
Wild-type	8.0 (6.95 - 9.36)	1
Y54C	25.0 (22.37 - 27.99)	3.1
L167F	41.6 (n.a. - 230.80)	5.2
Q192R	25.5 (17.88 - 53.94)	3.2

Table S9: Enzyme kinetics metrics of wt $3CL^{pro}$ and mutants with the substrate Ac-Abu-Tle-Leu-Gln↓MCA. V_{max}, maximal reaction rate; RFU, relative fluorescent units; K_m, Michaelis-Menten constant; k_{cat}, catalytic constant or turnover number.

	wt	Y54C	L167F	Q192R
V_{max} (RFU/min)	1.71	1.31	1.75	1.03
K_m	80.6	108.5	134.1	163.3
k_{cat} (min^{-1})	0.017	0.013	0.018	0.010
k_{cat}/K_m	1.78×10^{-4}	1.21×10^{-4}	1.31×10^{-4}	0.63×10^{-4}

Table S10: Nirmatrelvir IC_{50} values obtained from Fluorescence Resonance Energy Transfer (FRET) assays.

	Nirmatrelvir IC_{50} (µM)	Fold change (versus wt)
Wild-type	0.085 (0.06 - 0.12)	1
Y54C	0.26 (0.19 - 0.40)	3
L167F	0.38 (0.23 - 0.85)	4.5
Q192R	0.43 (0.33 - 0.59)	5

Table S11: Nirmatrelvir IC_{50} values obtained from recombinant SARS-CoV-2-mCherry.

	Nirmatrelvir IC_{50} (nM) 48 hpi	Fold change (versus wt)	Nirmatrelvir IC_{50} (nM) 72 hpi	Fold change (versus wt)
Wild-type	8.68 (7.85 - 9.61)	1	8.51 (7.68 - 9.44)	1
L167F	16.24 (14.31 - 18.42)	1.9	20.05 (17.71 - 22.70)	2.4
L167F F305L	20.07 (17.69 - 22.75)	2.3	23.10 (19.75 - 26.97)	2.7

Table S12: cloning primer for VSV vectors.

Name	Sequence (5'-3' direction)
33n-before-KpnI-for	GAACCGGTCCTGCTTTCACC
G-cut1-rev	CATTTTTCTAAAACCACTCTGCAAAACAGCTGAGGTGATCTTTCCA AGTCGGTTC
cut1-for	ATCACCTCAGCTGTTTTGCAG
cut2-L-rev	GTCGGTCTCAAAATCGTGGACTTCCATGATTGTTCTTTTCACTGCAC TTTG
cut2-L-for	AGTGCAGTGAAAAGAACAATCATGGAAGTCCACGATTTTGAG
33n-after-HpaI-rev	GATGTTGGGATGGGATTGGC

Table S13: cloning primer for 3CL^{pro}-Off and -On mutant variants.

Name	Sequence (5'-3' direction)
blasticidin-for	CATTCGATTAGTGAACGGATCTC
3CL^{pro} -L167F-for	GCACCATATGGAATTTCCAACTG
3CL^{pro} -L167F-rev	CATGAACTCCAGTTGGAAATTCC
3CL^{pro} -Y54C-for	CATGCTTAACCCTAATTGTGAAGATTTACTC
3CL^{pro} -Y54C-rev	CTTACGAATGAGTAAATCTTCACAATTAGGG
3CL^{pro} -F305L-for	GCTCAGGTGTTACTCTCCAAAG
3CL^{pro} -F305L-rev	CACTGCACTTTGGAGAGTAACAC
3CL^{pro} -Q192R-for	GTTGACAGGCAAACAGCACGAGCAGCTG
3CL^{pro} -Q192R-rev	GTCCGTACCAGCTGCTCGTGCTGTTTG
Omicron-for	CAATGTGCTATGAGGCACAATTTCAC
Omicron-rev	CTTAATAGTGAAATTGTGCCTCATAGC
3CL^{pro} -A194S-for	CAAACAGCACAAGCATCTGGTACG
3CL^{pro} -A194S-rev	GTGTCCGTACCAGATGCTTGTGC
3CL^{pro} -G138S-for	CCCAATTTCACTATTAAGAGTTCATTCCTTAATG
3CL^{pro} -G138S-rev	ACCACATGAACCATTAAGGAATGAACTCTTAATA
hygro-P-for	CTGTTTTGACCTCCATAGAAGATTCTAGAGCTAGCATGGATAATCT CACAAAAGTTC
P-hygro-rev	GAGGGAGAGGGGCGGATCCCCTTAATTAACTACAGAGAATATTTG ACTCTCGC

Data file S1: Sequence for SARS-CoV-2-mCherry. The non-annotated sequence of unmodified rWA1 SARS-CoV-2-mCherry is provided in the standard fasta (.fasta) format.

Data file S2: Accession numbers for SARS-CoV-2 mutants introduced into SARS-CoV-mCherry. Accession identifiers (IDs), collection and submission dates and location from the GISAID database are provided for L167F and F305L mutants.

Data file S3: Raw, individual-level data for experiments where n<20. The file provides raw and normalized data for all figures, which contain graphs generated in GraphPad Prism.

Data file S4: Sequence for VSV-3CL^{pro}. The annotated sequence of VSV-3CL^{pro} is provided in the standard GenBank (.gb) format.
